# Light chain skewing in autoantibodies and B-cell receptors of the citrullinated antigen-binding B-cell response in rheumatoid arthritis

**DOI:** 10.1371/journal.pone.0247847

**Published:** 2021-03-30

**Authors:** Linda M. Slot, Rochelle D. Vergroesen, Priscilla F. Kerkman, Ellen Staudinger, Sanne Reijm, Hugo J. van Dooren, Ellen I. H. van der Voort, Tom W. J. Huizinga, René E. M. Toes, Hans U. Scherer

**Affiliations:** Department of Rheumatology, Leiden University Medical Center, Leiden, The Netherlands; Duke University School of Medicine, UNITED STATES

## Abstract

Rheumatoid arthritis (RA) is a chronic autoimmune disease affecting 1% of the world population. RA is associated with the presence of autoantibodies, of which anti-citrullinated protein antibodies (ACPA) are most prominent. ACPA are produced by citrullinated antigen-binding B cells that have presumably survived tolerance checkpoints. So far, it is unclear how and when such autoreactive B cells emerge. Light chain (LC) rearrangement and mutation rates can be informative with regard to selection steps during B-cell development. Therefore, we studied LC characteristics of ACPA-expressing B cells and secreted ACPA with the aim to better understand the development of this disease-specific, autoreactive B-cell response. Paired ACPA-IgG and ACPA-depleted IgG were isolated from serum (n = 87) and synovial fluid (SF, n = 21) of patients with established RA. We determined the LC composition for each fraction by ELISA using kappa(Igκ)- and lambda(Igλ) LC-specific antibodies. Cellular LC expression was determined using flow cytometry. In addition, we used a B-cell receptor (BCR)-specific PCR to obtain LC variable region sequences of citrullinated antigen- and tetanus toxoid (TT)-binding B cells. In serum, we observed an increased frequency of lambda LC in ACPA-IgG (1.64:1) compared to control IgG (2.03:1) and to the κ/λ ratio reported for healthy individuals (2:1). A similar trend towards higher frequencies of lambda LCs was observed for ACPA-IgG in SF (1.84:1). Additionally, the percentage of Igλ-expressing B cells was higher for citrullinated antigen-binding B cells (51%) compared to TT-specific (43%) and total CD19^+^CD20^+^ B cells (36%). Moreover, an increased Igλ percentage was observed in BCR-sequences derived from ACPA-expressing (49%) compared to TT-specific B cells (34%). Taken together, we report an enhanced frequency of lambda LCs in the secreted ACPA-IgG repertoire and, on the cellular level, in BCR sequences of ACPA-expressing B cells compared to control. This skewing in the autoreactive B-cell repertoire could reflect a process of active selection.

## Introduction

The majority of rheumatoid arthritis (RA) patients harbor autoantibodies that recognize citrullinated proteins (commonly termed anti-citrullinated protein antibodies, ACPA). A hallmark of ACPA is their specificity for RA. ACPA can be detected before the onset of disease and are valuable biomarkers in clinical practice [1–3]. Interestingly, ACPA levels in serum of RA patients correlate with the frequency of citrullinated antigen-binding (ACPA-expressing) B cells in peripheral blood [4] and can reach levels similar to peak levels of protective antibody responses against recall antigens such as tetanus toxoid (TT) [1,2,5]. However, the avidity of ACPA is remarkably low compared to other antibody responses (e.g. against TT) despite a much higher somatic hypermutation rate [1,6–8]. Moreover, ACPA-IgG were found to be highly glycosylated in the variable domain and to be highly cross-reactive with other post-translational protein modifications [9–11]. This is intriguing, as it suggests that ACPA-expressing B cells deviate from the ‘conventional’ mechanisms of positive and negative selection and affinity maturation that are thought to govern the generation of high avidity, non-autoreactive clones, such as those observed against recall antigens [12]. Conventionally, such selection processes occur at various stages of B-cell development and lead to modifications of the B-cell receptor (BCR) aimed at minimizing autoreactivity, while maintaining a broad repertoire capable of mounting a protective immune response. Such modifications can affect both chains of the BCR, nevertheless, most studies on autoreactivity focus on the heavy chain. The alterations to the BCR can occur centrally during B-cell development in the bone marrow and in germinal centers (GC) or GC-like structures in the periphery. Understanding these processes in the context of human autoreactive B cells may be crucial to comprehend how ACPA-expressing B cells escape tolerance checkpoints and at what stage of B-cell development tolerance is breached.

BCR light chains (LCs) are initially generated in the bone marrow after successful rearrangement and expression of the heavy chain (HC). LC rearrangement starts on the immunoglobulin kappa (Igκ) locus, but when resulting in an unproductive rearrangement, it will lead to negative selection of the B cell in the bone marrow before entering the periphery. Likewise, LC rearrangement can result in an autoreactive BCR, which can also lead to negative selection by either apoptosis or anergy induction. Alternatively, B cells can rearrange the LC of the autoreactive BCR (receptor editing). The new LC can consist of V-genes positioned towards the 5’ end and/or J-genes positioned towards the 3’ end of the Igκ locus, can consist of V-genes on the second Igκ allele or of V-genes on one of the immunoglobulin lambda (Igλ) loci [13,14]. The characteristics of LCs are interesting in the context of selection, as the use of kappa or lambda V-genes and the LC mutation rate can be indicative of consecutive rearrangements during B-cell development. In addition, V-gene restriction may indicate structural requirements for antigen recognition [15–18].

Overall, the aforementioned processes result in an average κ/λ LC ratio of 2:1 for antibodies in serum of healthy individuals [19,20]. Notably, the probability of a B cell expressing Igλ increases with each round of BCR editing. Thus, a decreased κ/λ LC ratio in a particular BCR repertoire can indicate multiple rounds of BCR editing in the bone marrow to avoid the expression of autoreactive BCRs, thereby allowing B cells to escape negative selection.

Next to these central mechanisms, B cells can undergo secondary BCR rearrangement in the periphery (receptor revision), as has been reported in GC(-like) structures in the synovium of RA patients [21–23], in circulating human peripheral blood B lymphocytes [24], in leukaemia patients [25] and in murine B-cell lines and *in vivo* mouse models [26–28]. Similar to receptor editing, multiple rounds of receptor revision can lead to a decrease in the κ/λ LC ratio, presumably reflecting an attempt to escape from negative selection against autoreactivity.

Together, these considerations indicate that LC rearrangements in a given BCR repertoire can reflect selective pressure during (autoreactive) B-cell development. This makes such BCR rearrangements valuable sources of information in an effort to understand autoreactive B-cell responses. Here, we used multiple methods to investigate the κ/λ LC ratio in the ACPA-expressing B-cell repertoire to obtain insight in the BCR composition of autoreactive B cells that escaped tolerance checkpoints. Our results demonstrate an increased frequency of lambda LCs in ACPA-IgG isolated from serum and synovial fluid, an increased percentage of citrullinated antigen-binding B cells expressing the lambda LC as measured by flow cytometry and an increase in lambda LCs in BCR sequences derived from single cell- and pool-sorted citrullinated antigen-binding B cells.

## Methods

### Patients

Peripheral blood, serum and synovial fluid (SF) samples were obtained from patients diagnosed with ACPA-positive RA visiting the outpatient clinic of the Department of Rheumatology at Leiden University Medical Center (LUMC), Leiden, The Netherlands. Patients fulfilled the 2010 classification criteria for RA [29] at the time of diagnosis and gave written informed consent for sample acquisition. Patients treated with biological or B-cell depleting agents were excluded. This study was approved by the Institutional Review Board of the Leiden University Medical Center (approval number P17.151).

### ACPA purification using CCP2-coated beads

ACPA were purified from serum and SF of RA patients by antigen affinity chromatography using 2^nd^ generation cyclic citrullinated peptide (CCP2)-coated beads, as previously described [30]. In short, biotinylated CCP2 (0.5 mg) was added to 5 mL of Pierce NeutrAvidin Plus UltraLink slurry resin (Thermo Scientific). After washing, beads were loaded into a 96-well filter plate. Serum or SF samples (1:5 diluted) were added and incubated for 2 hours while shaking. ACPA bound to the resin were eluted by washing with 0.1M formic acid, followed by direct neutralization with 2M Tris buffer. The flow-through was obtained by centrifugation and one washing step, resulting in serum or SF ACPA-deleted control samples. CCP2 ELISAs were performed to validate ACPA purification in isolated ACPA and ACPA-depleted control samples. Samples with incomplete isolation/depletion were excluded from the analysis.

### ELISA of secreted antibodies in serum, synovial fluid and culture supernatants

The presence of light chains (LC) in ACPA-IgG was assessed by incubation of isolated ACPA or ACPA-depleted serum (n = 87 patients) and SF (n = 21 patients) on ELISA plates coated with mouse anti-human immunoglobulin lambda LC (Igλ; clone JDC-12, BD Pharmingen) or mouse anti-human immunoglobulin kappa LC (Igκ; clone G20-193, BD Pharmingen) monoclonal antibodies. ACPA bound by the respective anti-LC antibodies were detected with polyclonal rabbit anti-human IgG-HRP (DAKO). Human reference serum (Bethyl Laboratories) was used as standard in all ELISA experiments and for data normalization.

ACPA-Ig levels in citrullinated antigen-binding B-cell cultures were based on reactivity towards the CCP2-peptide. Biotinylated CCP2-peptides were coupled to streptavidin-coated ELISA plates, followed by incubation with 1:2 diluted supernatant from cultures. ACPA-Ig levels were detected with secondary antibodies for IgG (rabbit anti-human IgG-HRP, DAKO), IgM (goat anti-human IgM-HRP, Millipore) or IgA (goat anti-human IgA-HRP, Invitrogen) to identify Ig-isotypes. The presence of tetanus toxoid (TT)-specific antibodies in supernatants of TT-specific B-cell cultures was determined using TT-coated (Staten Serum Institute) plates. Detection of TT-specific antibodies was identical to Ig-detection of ACPA.

### Flow cytometry

Streptavidin tetramers were used to identify ACPA-expressing B cells as previously described [4]. Peripheral blood mononuclear cells (PBMCs) were isolated from 40 ml of peripheral blood by Ficoll-Paque gradient centrifugation and stained with Fixable Violet (405nm) Dead Cell Stain kit (Thermofisher), CD3 Pacific Blue (clone UCHT1, BD Pharmingen), CD14 Pacific Blue (clone M5E2, BD Pharmingen), CD19 APC-Cy7 (clone SJ25C1, BD Pharmingen), CD20 AlexaFluor 700 (clone 2H7, BD Pharmingen) and CD27 PE-Cy7 (clone M-T271, BD Pharmingen). Citrullinated antigen-binding B cells were identified by a double positive staining using two CCP2-containing tetramers (APC- and BV605-labelled) and negative staining for the arginine control variant (PE-labelled). Similarly, CD19^+^CD20^+^ double positive B cells were considered TT-specific if they stained double positive for TT-APC and TT-PE.

Furthermore, in flow cytometry experiments used to determine Igκ and Igλ frequencies on the cellular level, cells were additionally stained with Igκ VioGreen (clone IS1124D5, Miltenyi Biotec) and Igλ FITC (clone IS724C7, Miltenyi Biotec). In-depth gating strategy for determination of Igκ and Igλ expression can be found in S1 Fig.

For FACS-sort experiments to isolate citrullinated antigen- and TT-binding B cells for single cell culture and sequencing, the panel was extended with IgG BV510 (BD Horizon, clone G18-145) and IgD FITC (clone IA6-2, BD Pharmingen). ACPA-expressing B cells were sorted on a BD FACSAriaII/III flow cytometer as pools of ten cells/well for direct lysis (CD19^+^) or as one cell/well for culture (CD19^+^CD20^+^), TT-specific B cells were only sorted as one cell/well for culture (CD19^+^CD20^+^).

### Single cell cultures

Single cell-sorted citrullinated antigen-binding CD19^+^CD20^+^ B cells (obtained from n = 19 patients) and TT-binding B cells (obtained from n = 4 of 19 patients) were cultured in 96-wells flat bottom plates on a layer of irradiated mouse fibroblasts transfected with human CD40 ligand (CD40L L-cells, 70Gy irradiated, 1x10^4^ cells/well) in Iscove’s Modified Dulbecco’s Medium containing 8% heat-inactivated fetal calf serum (FCS), 100U/mL penicillin/streptomycin and 1.82 mM GlutaMax for 10–13 days. Additionally, this culture medium contained 1ng/ml IL-1β (R&D Systems), 500ng/ml R848 (Invivogen), 50ng/ml IL-21 (Gibco), 0.3ng/ml TNFα (R&D Systems), 20μg/ml IgG depleted Apotransferrin (Sigma-Aldrich) and 100–446 nM β-mercaptoethanol (Merck) as used previously [31].

### mRNA isolation and cDNA synthesis

Direct mRNA lysis was performed on pools of 10 CD19^+^ citrullinated antigen-binding B cells with a mixture of 0.2% Triton X-100 (Sigma) in ddH_2_O, RNase inhibitor (25 U, TaKaRa), Oligo-dT30VN (10 pmol, S1 Table, IDT) and dNTPs (10 nmol, ThermoFisher) per pool. mRNA from single cell cultures was isolated using TRIzol reagent following the manufacturer’s protocol (ThermoFisher). 300 μL of TRIzol was used for cell lysis and mRNA was precipitated overnight with isopropanol. Subsequently, Oligo-dT30VN (10 pmol) and dNTPs (10 nmol) were added. cDNA from both pool-isolated and single cell-isolated mRNA was synthesized using the following method: mRNA was incubated for 3 min. at 72°C. Subsequently, 5x first-strand buffer (TaKaRa), Betaine BioUltra (10 μmol, Sigma-Aldrich), DTT (10 nmol, TaKaRa), RNase inhibitor (10 U), SMARTScribe reverse transcriptase (50 U, TaKaRa), Template-Switching Oligo (TSO, 10 pmol, S1 Table, Exiqon) and RNAse-free water was added per sample. Samples were incubated for 90 min. at 42°C, followed by 10 cycles of 2 min. at 50°C and 2 min. at 42°C. cDNA synthesis was finalized by incubation for 15 min. at 72°C. cDNA of pool-sorted samples was pre-amplified by adding Phusion Flash High-Fidelity PCR Master Mix (1x, ThermoFisher), RNAse-free H_2_O and ISPCR (2.5 pmol, S1 Table, IDT) for a second PCR reaction. Samples were incubated for 2 min. at 98°C, followed by 23 cycles of 3 sec. at 98°C, 15 sec. at 67°C and 5 min. at 72°C, followed by a final extension for 5 min. at 72°C. Pre-amplified pool-sorted cDNA was purified using Agencourt AMPureXP magnetic beads (Beckman Coulter) following the manufacturer’s protocol, with exception of a 5:4 ratio of cDNA:beads and elution in 15 μL nuclease-free H_2_O.

### ARTISAN PCR

Immunoglobulin PCR products were amplified using an adapted protocol for low cDNA amount based on the Anchoring Reverse Transcription of Immunoglobulin Sequences and Amplification by Nested (ARTISAN) PCR [32]. 2 μL of cDNA was added to a combination of Phusion Flash High-Fidelity PCR Master Mix (0.5x), nuclease-free H_2_O, SA forward primer (5 pmol, SA.pcr) and either one of Ig-specific reverse primers (5 pmol, Cα.pcr, Cγ.pcr, Cμ.pcr, Cκ.pcr or Cλ.pcr). Mixtures were incubated for 2 min. at 98°C, followed by 40 cycles of 1 sec. at 98°C, 15 sec. at 69°C and 15 sec. at 72°C, and final extension for 1 min. at 72°C. Subsequently, each pool-sorted ARTISAN sample was barcoded by adding a mix of Phusion Flash High-Fidelity PCR Master Mix (1x), nuclease-free H_2_O, one of the SA forward barcode family primers (5 pmol, SA.bc) and one of the Ig-specific reverse barcode family primers (5 pmol, Cα.bc, Cγ.bc, Cμ.bc, Cκ.bc or Cλ.bc). Mixtures were incubated for 2 min. at 98°C, followed by 10 cycles of 1 sec. at 98°C, 15 sec. at 65°C and 15 sec. at 72°C, and final extension for 1 min. at 72°C. See S1 Table for sequences of ARTISAN and barcoding primers. All primers were purchased from IDT.

### BCR sequencing

ARTISAN products from single-sorted cells were used for Sanger sequencing on the Applied Biosystems 96-capillary (ABI3730xl) system. Barcoded products from pool-sorted cells were pooled in 1:1 ratios, purified and sequenced on the PacBio RSII system.

### Data analyses

Barcodes corresponding to different pools and donors were separated with Geneious 9.1.8. Both single- and pool-sorted sequences were analyzed with IMGT-(high)Vquest [33], ARGalaxy [34] and Microsoft Access to define B cell clones as unique productive sequences based on identical V-gene+J-gene+CDR3(AA) (S2 Table). Clones were identified to avoid bias caused by clonal expansion or differences in BCR mRNA expression in resting versus activated B cells. LC sequences obtained from single-sorted ACPA-expressing B cells were selected for all IgH isotypes (Ig_all_) or the IgG isotype only. Sequencing data from pool-sorted ACPA-expressing B cells contained LC paired with HC transcripts from IgG (83%), IgA (11%) and IgM (6%) cells, but direct HC-LC pairing is not compatible with the PacBio sequencing method. Statistical analyses were performed using GraphPad prism 8.0. Correlations were assessed as parametric paired correlations, One-way ANOVA or Chi square tests and corrected with Holm-Bonferroni for multiple comparisons. *P* Values <0.05 were considered significant.

## Results

### Lambda LC in secreted ACPA-IgG isolated from serum and SF

**Fig 1:**
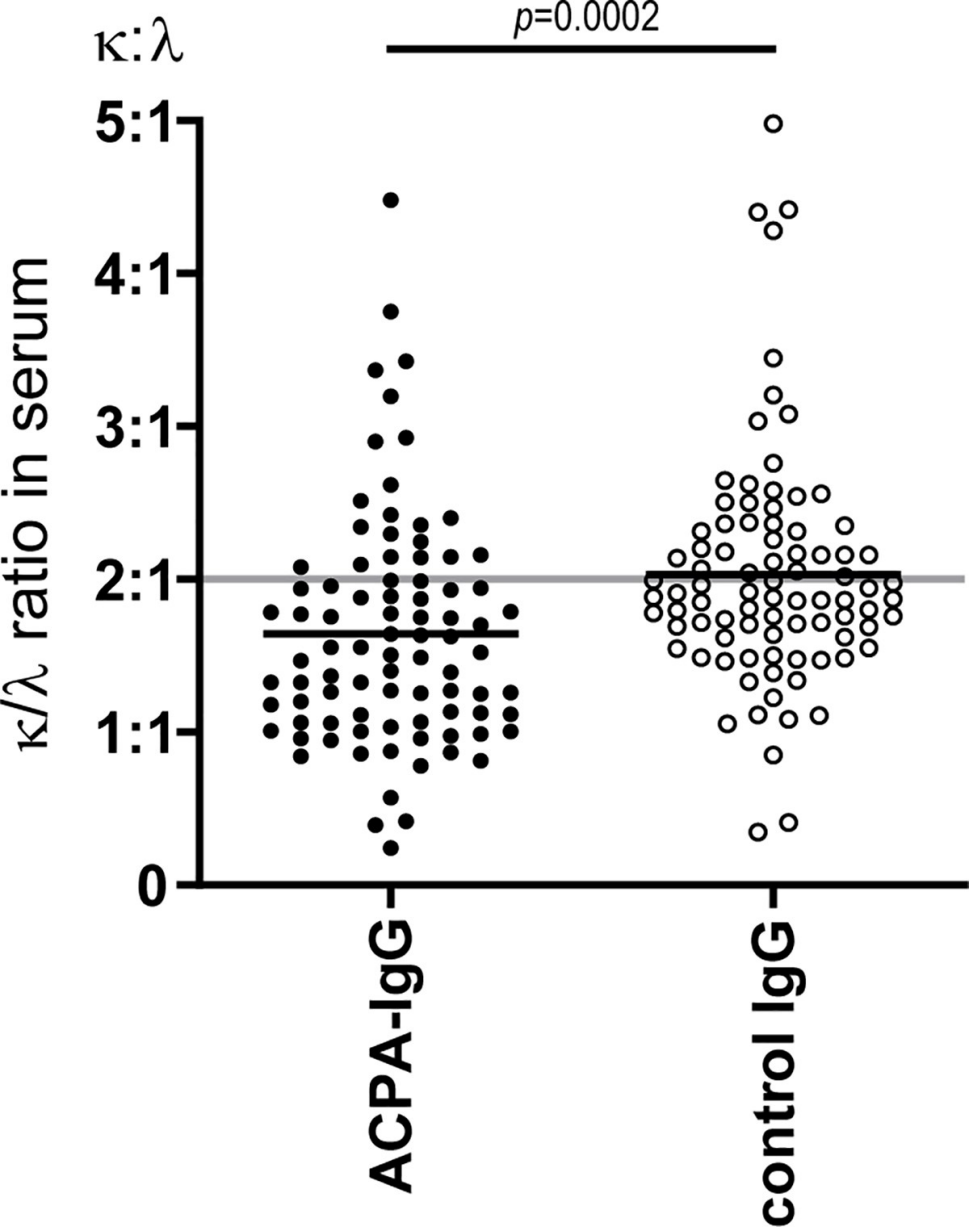
Frequency of lambda LC in ACPA-IgG and control IgG in serum of RA patients. κ/λ ratios in paired ACPA-IgG and control IgG in serum of RA patients (n = 87) where the grey line depicts the κ/λ ratio of 2:1 (67%-33%) in healthy individuals as reported in literature. Data were assessed as parametric paired samples in student’s *t*-test.

We first determined the frequency of lambda and kappa LCs of secreted ACPA-IgG in serum and synovial fluid. To this end, ACPA were isolated from serum of 87 RA patients using CCP2-coated beads, resulting in paired samples containing “ACPA-IgG” or “IgG depleted of ACPA” (control IgG) per patient. The average κ/λ ratio in control IgG was equal to 2.03 (SD 0.77, Fig 1) as expected for normal human serum (2:1) [19,20]. In contrast, the frequency of lambda LC was significantly increased in ACPA-IgG (mean κ/λ ratio 1.64, SD 0.75; *p* = 0.0002, Fig 1; Table 1). Additionally, ACPA-IgG were isolated from SF of 21 patients to study if the higher frequency of lambda LC was also present in this compartment. Indeed, a similar trend was observed in SF, with ACPA-IgG containing relatively more lambda LC (mean κ/λ ratio 1.84, SD 0.82; Table 1) than control IgG (mean κ/λ ratio 2.02, SD 0.65; Table 1), although this difference did not reach statistical significance.

**Table 1:** Summary of all κ/λ LC ratios observed in ACPA-expressing, TT-specific and total IgG/total CD19^+^CD20^+^ B cells as measured with ELISA, flow cytometry and BCR sequencing.

	**ACPA κ/λ**	**TT κ/λ**	**total κ/λ**
**Serum** (IgG)	1.64:1	-	2.03:1***
**SF** (IgG)	1.84:1	-	2.02:1
	**ACPA κ/λ (%)**	**TT κ/λ (%)**	**total κ/λ (%)**
**FACS** (Ig_all_)	49:51	64:36****	57: 43**
**Single cell seq.** (Ig_all_ cells)	51:49	66:34*	-
**Single cell seq.** (IgG cells)	52:48	64:36	-
**Single cell seq.** (Ig_all_ clones)	54:46	69:31	-
**Single cell seq.** (IgG clones)	53:47	67:33	-
**Pool seq.** (Ig_all_)	63:37	-	-

Statistical tests resulting in significant differences between ACPA and TT data or ACPA and total IgG/total CD19^+^CD20^+^ B cells data are depicted with level of significance in the column of the group to which ACPA was compared.

IgG = immunoglobulin G.

Ig_all_ = all immunoglobulin isotypes (IgA, IgG or IgM).

SF = synovial fluid.

FACS = flow cytometry.

seq = sequencing.

scSeq = single cell sequencing.

ACPA = anti-citrullinated protein antibodies.

TT = tetanus toxoid.

total = control; total IgG in ELISA or total CD19^+^CD20^+^ B cells in FACS.

dash (-) = not determined.

level of significance; *p* ≤ 0.05 = *, *p* ≤ 0.01 = **, *p* ≤ 0.001 = ***, *p* < 0.0001 = ****.

### Igκ and Igλ expression on B cells

**Fig 2:**
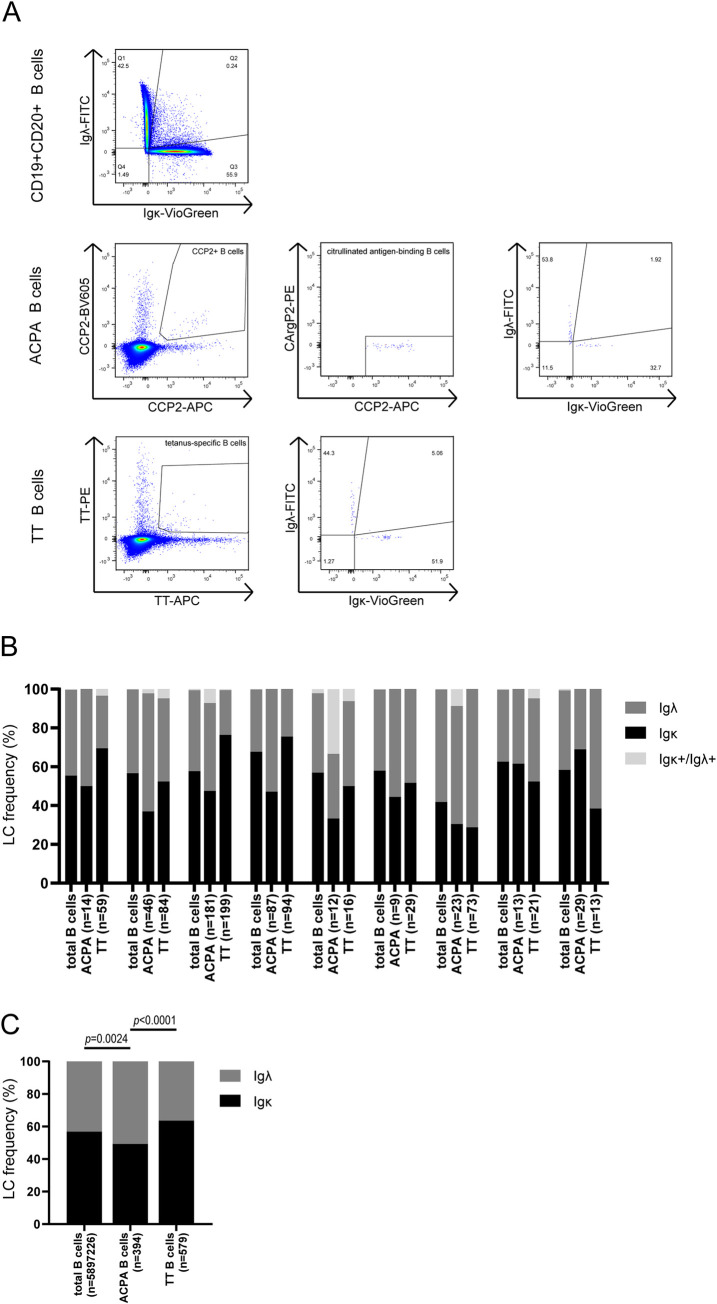
Igκ and Igλ expression by total CD19^+^CD20^+^, citrullinated antigen- and TT-binding B cells measured with flow cytometry. A) CD19^+^CD20^+^ double positive B cells were gated as Igκ- or Igλ-expressing B cells based on isotype controls. Cells were defined as citrullinated antigen-binding B cells when cells were double positive for CCP2-APC and CCP2-BV605 tetramers, while negative for CArgP2-PE control. Cells were defined as TT-specific B cells when double positive for both TT-PE and TT-APC tetramers. B) LC expression by total CD19^+^CD20^+^ B cells, citrullinated antigen-binding B cells and TT-specific B cells in RA patients (n = 9). A low frequency of double negative (Igκ^-^/Igλ^-^) cells were excluded from this analysis as their LC expression could not be determined with certainty. C) Frequency of LC expression based on the total number of cells expressing Igκ or Igλ from data of 9 RA donors depicted in B. Double negative (Igκ^-^/Igλ^-^) and double positive (Igκ^+^/Igλ^+^) cells were excluded from this analysis. Data were analyzed with the Chi square test and Holm-Bonferroni for multiple comparisons.

Next, we investigated whether the increased lambda LC frequency present in secreted ACPA-IgG was related to antibody production by Igλ-expressing plasma cells, or whether an overall increase in Igλ expression by citrullinated antigen-binding B cells could be detected. We studied the Igκ and Igλ expression in citrullinated antigen-binding and control B cells (i.e. TT-specific B cells and total CD19^+^CD20^+^ B cells) using flow cytometry (Fig 2A).

The number of analyzed cells varied between donors: 9–181 (median 23) for citrullinated antigen-binding B cells, 13–199 (median 59) for TT-specific B cells and 182,980–1,962,822 (median 556,826) for total CD19^+^CD20^+^ B cells. Seven out of nine donors showed a similar pattern with an enhanced Igλ frequency in citrullinated antigen-binding B cells compared to total CD19^+^CD20^+^ B cells. In six out of these seven donors, citrullinated antigen-binding B cells expressed Igλ more frequently than TT-specific control cells (Fig 2B; Table 1). Furthermore, when absolute numbers of CD19^+^CD20^+^, citrullinated antigen- and TT-binding cells of all 9 donors were pooled and analyzed for LC expression, a significant increase in Igλ-positive B cells was observed for citrullinated antigen-binding B cells compared to total CD19^+^CD20^+^ B cells (*p* = 0.0024) and TT-specific B cells (*p<*0.0001). TT-specific B cells showed a higher frequency of Igκ compared to total CD19^+^CD20^+^ cells (Fig 2C). Additionally, 4.8% of citrullinated antigen-binding B cells expressed double positive Igκ/Igλ BCRs, (observed in four out of nine RA patients) compared to 1.5% of TT-specific B cells (observed in five out of nine RA patients) and 0.5% of total CD19^+^CD20^+^ B cells (observed in all donors).

In summary, an enhanced frequency of Igλ in citrullinated antigen-binding B cells was observed compared to total and TT-specific B cells from the same patients, although this was not the case for all donors.

### Frequency of Igκ and Igλ in BCR sequences

**Fig 3:**
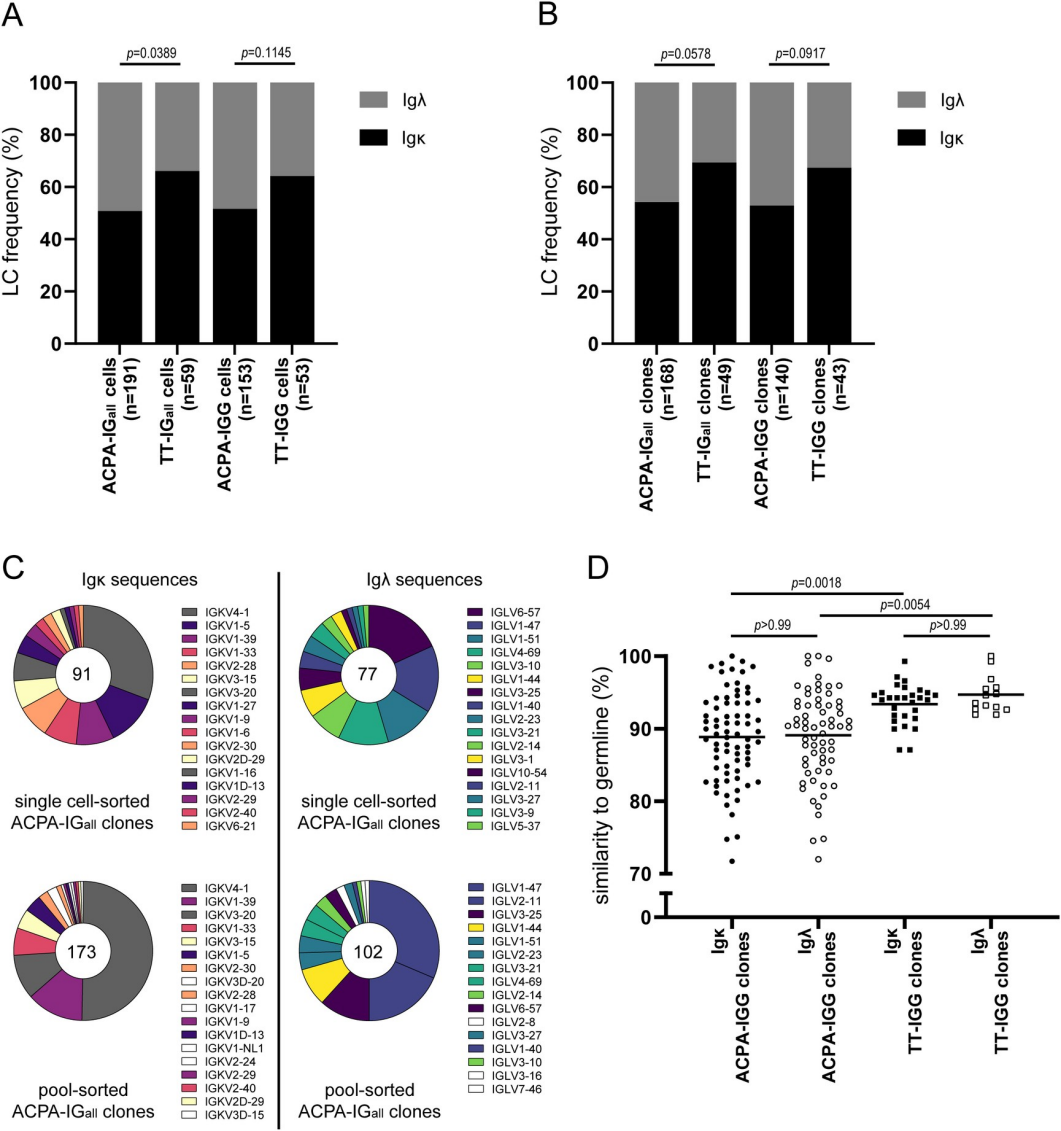
Igκ and Igλ frequency and characteristics in BCR sequences of citrullinated antigen- and TT-binding B cells. CD19^+^CD20^+^ citrullinated antigen-binding B cells (derived from 19 patients) or TT-binding B cells (derived from 4 out of 19 patients) were single cell FACS-sorted and cultured for 10–13 days. Single cell-derived Ig_all_ and IgG sequences were analyzed for Igκ and Igλ frequency based on sequences from A) all single cells and B) individual clones (defined as identical V-gene+J-gene+CDR3(AA)) to exclude bias due to clonal expansion. Data were statistically analyzed with Chi square test and Holm-Bonferroni correction for multiple comparisons. C) V-gene repertoire of single cell- and pool-sorted ACPA-Igκ clones (left panels) and ACPA-Igλ clones (right panels). V-genes, which were not present in single cell-sorted ACPA-LC clones, were colored white in the pie charts of pool-sorted ACPA-LC clones. D) Mutation rate of Igκ and Igλ sequences in citrullinated antigen- and TT-binding B cells. Data was statistically analyzed with One-way ANOVA and Holm-Bonferroni correction for multiple comparisons.

Next, we studied LC sequences obtained from citrullinated antigen- and TT-binding B cells to determine whether the increased Igλ expression observed with flow cytometry was due to the expansion of individual Igλ expressing B-cell clones. To this end, citrullinated antigen-binding CD19^+^CD20^+^ B cells from 19 patients were sorted one cell per well and expanded *in vitro* for 10–13 days. In addition, TT-binding CD19^+^CD20^+^ B cells from 4 of these 19 patients were sorted and cultured using the identical protocol. Antibodies in the supernatant of cultured cells were tested with ELISA to determine CCP2- or TT-reactivity. Subsequently, cell lysis, cDNA synthesis, specific BCR amplification and Sanger sequencing were performed. This resulted in LC sequences of 191 citrullinated antigen- and 59 TT-binding B cells (Ig_all_), of which 153 and 59 were of the IgG isotype, respectively. In both Ig_all_- and IgG-(sub)datasets, ACPA-expressing B cells contained a higher frequency of Igλ (49.2% and 48.4%, respectively) compared to TT-specific B cells (33.9% and 35.8% respectively; Fig 3A; Table 1). These frequencies corresponded to the Igλ expression observed by flow cytometry. Noteworthy, 5 citrullinated-antigen binding cells, of which 3 had the IgG isotype, contained productive sequences for both Igκ and Igλ (2.7% and 2.0% respectively), whereas no double LC sequences were observed for TT-specific cells in either dataset.

To identify clonal expansion, sequences were analyzed for their V-gene, J-gene and the CDR3 region in amino acids (AA). Cells containing an identical V-gene, J-gene and CDR3(AA) were defined as one clone. This resulted in 168 ACPA clones and 49 TT clones when analyzing the Ig_all_ dataset and in 140 ACPA clones and 43 TT clones when analyzing the IgG subset (Fig 3B; Table 1). A trend towards a higher frequency of Igλ in ACPA clones (45.8% and 47.1% for Ig_all_ and IgG, respectively) compared to TT clones (30.6% and 32.6% for Ig_all_ and IgG, respectively) was observed when clonal expansions were taken into account. Additionally, the 5 ACPA cells, which contained productive sequences for both Igκ and Igλ, were also observed in the clone-corrected data (3.1% and 2.2%, respectively). To exclude potential bias due to the single cell culture approach, we also assessed pools of ACPA-expressing B cells by next generation sequencing. While no distinction of LCs coupled to IgG, IgA or IgM could be made in this experimental setup, we observed a similar trend for the LC distribution as for the single cell-sorted dataset (37.2% Igλ versus an expected Igλ frequency of 33% (Table 1)).

Altogether, the sequencing analyses demonstrated that the BCR of citrullinated antigen-binding B cells was more frequently composed of a Igλ than a Igκ LC compared to BCRs derived from TT-control cells. Furthermore, the results showed that this increase in Igλ was not related to clonal expansion. To determine if specific Igκ variable region (IGKV) genes or Igλ variable region (IGLV) genes were common in citrullinated antigen-binding B-cell clones across sequencing methods, we assessed V-region frequencies in Ig_all_ single cell-sorted LC clones and compared these to V-region frequencies in Ig_all_ pool-sorted LC clones. A similar V-gene pattern was observed for both Igκ and Igλ sequences across the different methods (Fig 3C). Intruigingly, IGKV4-1 was predominantly found in both single cell-sorted LC clones (31%) and pool-sorted LC clones (50%). Similarly, IGKV1-39, IGKV1-33, IGKV3-15 and IGKV3-20 were substantially represented in both ACPA datasets. Although frequencies of some IGLV-genes were different between the datasets (e.g. IGLV6-57, IGLV3-10 and IGLV2-11), 13 of 17 IGLV-genes were discovered in both datasets. In contrast to the predominantly expressed IGKV4-1 in ACPA-IgK clones, a dominant IGLV-gene could not be observed in ACPA-Igλ clones. However, IGLV1-47 was frequently found among IGLV-genes in single cell-sorted ACPA-Igλ clones (16%) and in pool-sorted ACPA-Igλ clones (31%). Altogether, these data indicate a diverse LC repertoire in BCRs of citrullinated antigen-binding B cells with a frequent presence of IGKV4-1 in ACPA-Igκ clones.

Finally, we determined the mutation rate in LC sequences of citrullinated antigen- and TT-binding B cell clones. We exclusively analyzed LCs obtained with single cell sequencing that were paired with IgG-isotype HCs. No significant differences were observed between Igκ and Igλ mutation rates within either antigen-specific BCR repertoire. Nevertheless, both Igκ and Igλ sequences derived from ACPA-expressing B cells contained more mutations (were less similar to germline) than their TT-specific counterparts (Fig 3D).

To summarize, the higher lambda LC frequency observed in serum and on the cellular level was supported by an increase in lambda LCs in sequences corrected for clonal expansion. Pool-sorted ACPA B-cell clones showed a similar trend towards an increased Igλ frequency as clones obtained with single cell cultures, excluding survival bias within the cell cultures. Lastly, our sequence data showed diverse Igκ and Igλ repertoires, with a high frequency of IGKV4-1 in ACPA-Igκ clones, and no significant difference in the mutation rate of Igκ and Igλ in ACPA-LC clones.

## Discussion

To prevent autoimmunity, the rearrangement of antibody light chains is an important mechanism by which B cells can modify autoreactive B-cell receptors during different stages of B-cell development and maturation. Autoreactive B cells emerge as drivers of autoimmune diseases despite such control mechanisms for reasons that are incompletely understood. Notably, the composition and sequence characteristics of antibody LCs can provide insights in the developmental steps and selection processes that autoreactive B cells have been exposed to. Here, we investigated the LC characteristics of ACPA-expressing B cells and in the secreted ACPA-IgG repertoire. We report that serum ACPA-IgG contain a higher frequency of lambda LCs compared to paired control IgG. A similar trend was observed in synovial fluid of RA patients and the same skewed LC pattern was observed on the cellular and molecular level in citrullinated antigen-binding B cells. Together, these data indicate that ACPA-expressing B cells develop under conditions that favor LC rearrangements leading to the expression of genes of the lambda locus.

The κ/λ LC ratio of the antibody repertoire in healthy individuals averages 67%-to-33%, which is in line with the results of control IgG in our data [19,20]. A skewing in LCs has been described for several diseases. A higher κ/λ LC ratio, i.e. a predominance of Igκ, has been observed for anti-granulocyte colony-stimulating factor antibodies [35], non-specific IgG antibodies in mixed connective tissue disease [36] and for CD10^+^ B cells in Hashimoto thyroiditis (HT) [37]. In contrast, a skewing towards Igλ has been described for anti-neutrophil antibodies [38], thyroid-stimulating autoantibodies [39], anti-lamin B antibodies [40] and circulating immune complexes in juvenile idiopathic arthritis (JIA) [41]. As the composition of the autoantibody repertoire in serum likely reflects the composition of the autoantibody secreting B-cell compartment (i.e. plasmablasts/plasma cells) [4,42], the predominance of Igλ in the secreted ACPA-IgG repertoire could be explained by selective survival and/or expansion of Igλ-antibody producing B-cell clones. Alternatively, it could be inherent to the citrullinated antigen-binding B-cell repertoire, irrespective of the maturation stage of the B-cell response. Our flow cytometry results support the latter, as the increased Igλ frequency was also detectable in citrullinated antigen-binding B cells (predominantly memory B cells). To further exclude the preferential expansion of Igλ-expressing B-cell clones, we analyzed BCR sequences derived from single cell-cultured ACPA-expressing B cells. These sequences showed a similar trend towards increased Igλ frequency, complementary to our observations by flow cytometry and the analysis of secreted ACPA-IgG. When corrected for clonality, based on the definition that cells with an identical V-gene, J-gene and CDR3 AA sequence belong to the same clone, the skewing towards a higher Igλ frequency remained. Importantly, we did not observe a similar phenomenon for TT-specific B cells in the same patients. Finally, to exclude bias due to differential B-cell survival *in vitro*, we performed next generation sequencing of pool-sorted ACPA-expressing B cells, which showed a similar trend. Together, these results support the notion that the increased Igλ frequency observed for ACPA is not due to clonal expansion of individual B cells, but rather that citrullinated antigen-binding B cells express Igλ more frequently than their non-autoreactive counterparts.

To our knowledge, this is the first in-depth analysis of the LC repertoire of ACPA-expressing B cells in RA. Previously reported studies focussed on either small numbers of monoclonal antibodies and their functional features [43–45], or did not report sufficient data on LC frequency to perform comparative analyses [46].

Predominance of Igλ in the ACPA BCR repertoire suggests that certain IGLV-genes or V-gene characteristics could facilitate the recognition of citrullinated antigens. Therefore, we analyzed V-gene frequencies in ACPA-Igκ and -Igλ clones. IGKV- and IGLV-gene patterns were similar for both our sequencing approaches and displayed a diverse repertoire. Intriguingly, IGKV4-1 was observed in both ACPA-Igκ datasets as a dominant IGKV-gene, whereas this V-gene is not as commonly present in non-specific Igκ repertoires [47,48]. Notably, the high frequency of IGKV4-1 in ACPA-Igκ clones observed by us is supported by the high prevalence of IGKV4-1 in sequences derived from citrullinated fillagrin (CFC1)/α-enolase (CEP-1)-binding B cells described by Titcombe *et al*. [46]. In the Igλ repertoire we observed a higher frequency of IGLV1-47 in both single cell- and pool-sorted ACPA-Igλ datasets (16% and 31%, respectively), compared to IGLV1-47 expression reported for B cells derived from human cord blood (6%) and normal controls (~10%) [49,50]. Despite these enhanced frequencies for certain V-genes, our findings do not show a highly restricted LC repertoire as observed in mice studies on certain antigen-specific B-cell responses (e.g. anti-p-azophenylarsonate (Ars) antibodies [15,16] and anti-Haemophilus influenzae type b capsular polysaccharide (Hib PS) antibodies [17,18]). In addition, no specific LCs have been associated with multiple autoimmune diseases in human, in contrast to selected heavy chain V-genes (e.g. IGHV4-34, previously called IGHV4-21, and IGHV3-23 [51–58]). More structural analyses would be needed to study the importance of LCs with IGKV4-1 in the recognition of citrullinated antigens and its role in a developing ACPA response.

Our data do not allow us to specifically determine whether the increased Igλ frequency in ACPA-expressing B cells is due to enhanced receptor editing in the bone marrow, receptor revision in the periphery or another unknown process. Considering receptor revision in the periphery, a relatively ‘late’ event in B-cell maturation, rearrangement of the LC would presumably be preceeded by various passages of ACPA-expressing B cells through GCs. In this case, it would be conceivable that kappa LCs show more extensive somatic hypermutation than lambda LCs. Interestingly, our data showed no difference in the somatic hypermutation rate between Igκ and Igλ sequences, suggesting that the increased Igλ frequency is not the result of receptor revision in the periphery but occurs earlier in B-cell development.

To understand when this divergent LC composition is generated, the κ/λ ratio of the LC repertoire of ACPA IgM-expressing B-cell clones should be analyzed in comparison to the LC repertoire of ACPA-IgG B-cell clones. However, feasibility is limited given the very low frequency of such cells in the periphery and the limitations associated with obtaining bone marrow-derived ACPA-expressing B cells. Nonetheless, based on our data, we would expect the IgM- or naïve compartment to show a similar skewing of the LC repertoire. This would be in line with the idea that the shift towards Igλ occurs early in the B-cell development.

Finally, our flow cytometry results indicate that 5% of citrullinated antigen-binding B cells contain both Igκ and Igλ compared to 0.5–1.5% of dual LC-expressing clones in total CD19^+^CD20^+^ and TT-specific B cells in RA patients, and compared to the range of 0.2–0.5% reported for healthy individuals [59,60]. Dual LC expression has been described abundantly in SLE. In mouse studies, B cells with dual Igκ^+^/Igκ^+^ LC expression were more frequently anti-DNA reactive. Additionally, studies in SLE patients reported frequencies of dual Igκ^+^/Igλ^+^ expression of up to 28% [61,62]. Allelic inclusion, the expression of BCRs with two different LCs, has been suggested as a way by which autoreactive B cells escape negative selection by “diluting” their autoreactive BCR with a second, non-autoreactive BCR to decrease signaling by autoantigens and increase the threshold for apoptosis [63,64]. To what extent this mechanism could also be relevant for ACPA-expressing B-cell maturation remains unknown.

In conclusion, we provide evidence for a skewing towards lambda LCs in secreted ACPA-IgG isolated from serum and synovial fluid, in surface expression on citrullinated antigen-binding B cells and in the BCR repertoire of these cells. Our data indicate that citrullinated antigen-binding B cells are under selective pressure, which may lead to LC rearrangements to avoid clonal deletion. Whether defective negative selection mechanisms are at play or whether citrullinated antigen-binding B cells receive additional survival signals that inhibit this deletion are interesting aspects of further investigations.

## Supporting information

S1 Fig In-depth gating strategy for determination of Igκ and Igλ expression in CD19+CD20+, TT-binding and citrullinated antigen-binding B cells. All subsets were gated on lymphocytes, single cells and CD19 (while negative for dead cell stain kit, CD3 and CD14). Subsequently, B cells were gated on CD19^+^CD20^+^ for total B cells control and antigen-specific B-cell subsets. B cells were defined as tetanus toxoid (TT)-binding B cells when CD19^+^CD20^+^ cells stained double positive for TT-APC and TT-PE. B cells were defined as citrullinated antigen-binding B cells when CD19^+^CD20^+^ cells stained double positive for CCP2-APC and CCP2-BV605 while negative for CArgP2 control.
(DOCX)

S1 Table Overview of primer sequences. (DOCX)

S2 Table LC data derived from citrullinated antigen- and tetanus-binding B cells. (PDF)
