# Supplementary figures and images for: Light chain skewing in autoantibodies and B-cell receptors of the citrullinated antigen-binding B-cell response in rheumatoid arthritis
Source: PLoS One. 2021 Mar 30;16(3):e0247847. doi: 10.1371/journal.pone.0247847 (PMC8009422; doi:10.1371/journal.pone.0247847)

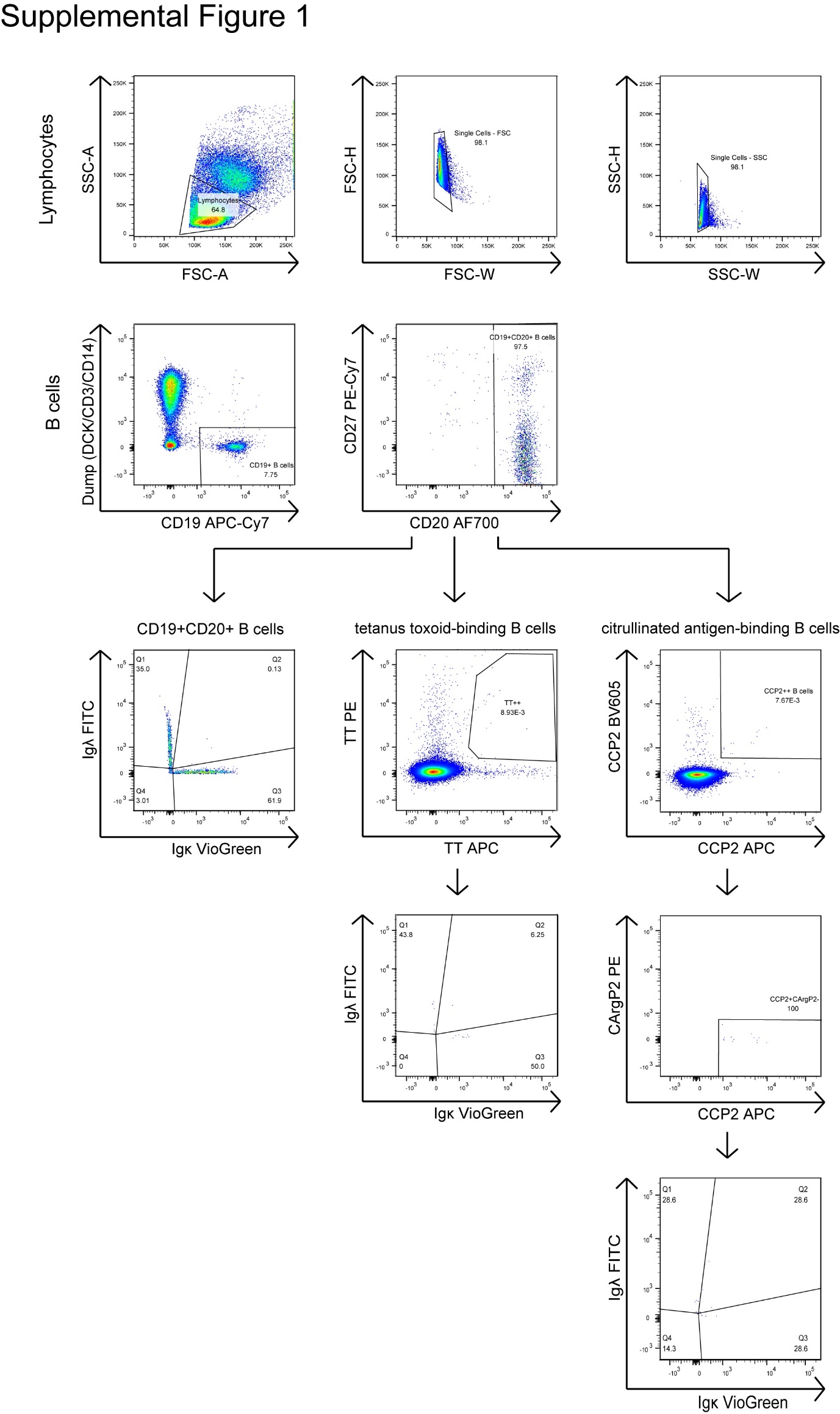

Supplement: S1 Fig — All subsets were gated on lymphocytes, single cells and CD19 (while negative for dead cell stain kit, CD3 and CD14). Subsequently, B cells were gated on CD19+CD20+ for total B cells control and antigen-specific B-cell subsets. B cells were defined as tetanus toxoid (TT)-binding B cells when CD19+CD20+ cells stained double positive for TT-APC and TT-PE. B cells were defined as citrullinated antigen-binding B cells when CD19+CD20+ cells stained double positive for CCP2-APC and CCP2-BV605 while negative for CArgP2 control. (DOCX) [file pone.0247847.s001.docx]
