# Supplementary material for: Light chain skewing in autoantibodies and B-cell receptors of the citrullinated antigen-binding B-cell response in rheumatoid arthritis
Source: PLoS One. 2021 Mar 30;16(3):e0247847. doi: 10.1371/journal.pone.0247847 (PMC8009422; doi:10.1371/journal.pone.0247847)
Supplement: S1 Table — (DOCX) [file pone.0247847.s002.docx]

S1 Table. Overview of primer sequences.

| **Primer** | **Sequence (5' to 3')** | **Protocol** |
| --- | --- | --- |
| Oligo-dT30VN | AAGCAGTGGTATCAACGCAGAGTACT30VN | cDNA synthesis |
| TSO | AAGCAGTGGTATCAACGCAGAGTACATrGrG+G | cDNA synthesis |
| ISPCR | AAGCAGTGGTATCAACGCAGAGT | pre-amplification PCR |
| SA.pcr | CTTAAGCAGTGGTATCAACGCAGAGTACGCG | ARTISAN PCR |
| Cα.pcr | CGCTCCAGGTCACACTGAGTGG | ARTISAN PCR |
| Cγ.pcr | GGAAGGTGTGCACGCCGCTGGTC | ARTISAN PCR |
| Cμ.pcr | CACAGGAGACGAGGGGGAAAAGGG | ARTISAN PCR |
| Cκ.pcr | CTGATGGGTGACTTCGCMG | ARTISAN PCR |
| Cλ.pcr | CACACYAGTGTGGCCTTGTTGGCTTG | ARTISAN PCR |
| SA.bc | **[F01-F12 bc]**CTTAAGCAGTGGTATCAACGCAGAGTACG | barcode PCR |
| Cα.bc | **[R01-R04 bc]**GCGGGAAGACCTTGGGG | barcode PCR |
| Cγ.bc | **[R01-R04 bc]**AGTAGTCCTTGACCAGGCAGCC | barcode PCR |
| Cμ.bc | **[R01-R04 bc]**GTTGGGGCGGATGCACTCC | barcode PCR |
| Cκ.bc | **[R01-R04 bc]**CTGCTTTGCTCAGCGTCAGG | barcode PCR |
| Cλ.bc | **[R01-R04 bc]**TGTTGGCTTGRAGCTCCTCAG | barcode PCR |
| R01 | CCATCTCATATGTAGTACTCT | Reverse (Cx.bc) barcodes |
| R02 | CCATCGCGATCTATGCACACG | Reverse (Cx.bc) barcodes |
| R03 | CCATCTGCAGTCGAGATACAT | Reverse (Cx.bc) barcodes |
| R04 | CCATCGACTCTGCGTCGAGTC | Reverse (Cx.bc) barcodes |
| F01 | GGTAGGCGCTCTGTGTGCAGC | Forward (SA.bc) barcodes |
| F02 | GGTAGTCATGAGTCGACACTA | Forward (SA.bc) barcodes |
| F03 | GGTAGTATCTATCGTATACGC | Forward (SA.bc) barcodes |
| F04 | GGTAGATCACACTGCATCTGA | Forward (SA.bc) barcodes |
| F05 | GGTAGACGTACGCTCGTCATA | Forward (SA.bc) barcodes |
| F06 | GGTAGTGTGAGTCAGTACGCG | Forward (SA.bc) barcodes |
| F07 | GGTAGAGAGACACGATACTCA | Forward (SA.bc) barcodes |
| F08 | GGTAGCTGCTAGAGTCTACAG | Forward (SA.bc) barcodes |
| F09 | GGTAGAGCACTCGCGTCAGTG | Forward (SA.bc) barcodes |
| F10 | GGTAGTCATGCACGTCTCGCT | Forward (SA.bc) barcodes |
| F11 | GGTAGAGAGCATCTCTGTACT | Forward (SA.bc) barcodes |
| F12 | GGTAGCGCATCGACTACGCTA | Forward (SA.bc) barcodes |
