# Supplementary material for: Light chain skewing in autoantibodies and B-cell receptors of the citrullinated antigen-binding B-cell response in rheumatoid arthritis
Source: PLoS One. 2021 Mar 30;16(3):e0247847. doi: 10.1371/journal.pone.0247847 (PMC8009422; doi:10.1371/journal.pone.0247847)
Supplement: S2 Table — (PDF) [file pone.0247847.s003.pdf]

Donor	Sequence	IgK/IgL	V-GENE and allele	V-REGION identity %	J-GENE and allele	J-REGION identity %	AA JUNCTION
Single cell-sorted ACPA-LC cells							
2	108	IgK	Homsap IGKV1-33*01 F	98.21	Homsap IGKJ2*02 F	97.22	CQQYDHVRTF
2	152	IgK	Homsap IGKV1-39*01 F	80.8	Homsap IGKJ4*01 F	84.21	CQKSQGALAF
2	174	IgK	Homsap IGKV2-40*01 F	89.56	Homsap IGKJ4*01 F	91.89	CMQRLRFPLTF
2	79	IgK	Homsap IGKV3-15*01 F	96.06	Homsap IGKJ2*01 F	89.47	CQQYNDWPVTF
2	80	IgK	Homsap IGKV3-15*01 F	96.06	Homsap IGKJ2*01 F	89.47	CQQYNDWPVTF
2	149	IgK	Homsap IGKV4-1*01 F	86.53	Homsap IGKJ4*01 F	80.0	CQHHFGMPPTF
2	100	IgK	Homsap IGKV4-1*01 F	85.86	Homsap IGKJ4*02 F	84.21	CQQHYDVPRTF
2	144	IgK	Homsap IGKV4-1*01 F	87.21	Homsap IGKJ3*01 F	92.11	CQQYYSVPFTF
2	136	IgK	Homsap IGKV4-1*01 F	83.84	Homsap IGKJ3*01 F	84.21	CQQYSGVPVTF
2	99	IgK	Homsap IGKV4-1*01 F	84.85	Homsap IGKJ1*01 F	83.33	CQQYYDVPRTF
2	145	IgK	Homsap IGKV4-1*01 F	81.48	Homsap IGKJ3*01 F	84.21	CQQYSGIPVTF
2	41	IgL	Homsap IGLV10-54*01 F	98.6	Homsap IGLJ3*02 F	100.0	CSAWDNSLSAWVF
2	54	IgL	Homsap IGLV2-14*01 F	99.65	Homsap IGLJ1*01 F	97.22	CSSYTSSSTQVF
2	183	IgL	Homsap IGLV3-9*01 F	100.0	Homsap IGLJ2*01 F	100.0	CQVWDSSTAYVVF
2	30	IgL	Homsap IGLV4-69*01 F	97.28	Homsap IGLJ2*01 F	97.37	CQTWGTDTVVF
2	31	IgL	Homsap IGLV4-69*01 F	97.28	Homsap IGLJ2*01 F	97.37	CQTWGTDTVVF
2	37	IgL	Homsap IGLV4-69*01 F	96.94	Homsap IGLJ2*01 F	96.15	CQTWGTYTVVF
2	33	IgL	Homsap IGLV4-69*01 F	97.28	Homsap IGLJ2*01 F	97.37	CQTWGTDTVVF
2	34	IgL	Homsap IGLV4-69*01 F	97.28	Homsap IGLJ2*01 F	97.37	CQTWGTDTVVF
2	35	IgL	Homsap IGLV4-69*01 F	97.28	Homsap IGLJ2*01 F	97.37	CQTWGTDTVVF
2	36	IgL	Homsap IGLV4-69*01 F	97.28	Homsap IGLJ2*01 F	97.37	CQTWGTDTVVF
2	38	IgL	Homsap IGLV4-69*01 F	97.28	Homsap IGLJ2*01 F	97.37	CQTWGTDTVVF
2	32	IgL	Homsap IGLV4-69*01 F	97.28	Homsap IGLJ2*01 F	97.37	CQTWGTDTVVF
2	18	IgL	Homsap IGLV6-57*01 F	93.79	Homsap IGLJ2*01 F	92.11	CQSYDTANHVLF
2	16	IgL	Homsap IGLV6-57*01 F	94.85	Homsap IGLJ2*01 F	94.74	CQSYDTTNHVLF
2	17	IgL	Homsap IGLV6-57*01 F	94.85	Homsap IGLJ2*01 F	92.11	CQSYDTTNHVLF
2	3	IgL	Homsap IGLV6-57*02 F	84.19	Homsap IGLJ3*02 F	94.59	CQSYYRGDWVL
6	109	IgK	Homsap IGKV1-33*01 F	98.57	Homsap IGKJ4*01 F	89.47	CQQYDNLLGTF
6	104	IgK	Homsap IGKV1-39*01 F	89.96	Homsap IGKJ2*01 F	81.58	CQQSYSTPYTF
6	81	IgK	Homsap IGKV3-15*01 F	100.0	Homsap IGKJ1*01 F	100.0	CQQYNNWPRTF
6	71	IgL	Homsap IGLV1-47*01 F	90.53	Homsap IGLJ3*02 F	97.14	CAAWDDDLGGPVF
6	63	IgL	Homsap IGLV1-47*01 F	91.23	Homsap IGLJ3*02 F	94.29	CAAWDDDLGGPVF
7	124	IgK	Homsap IGKV1-33*01 F	87.81	Homsap IGKJ4*01 F	94.74	CQEYDNLGLSF
7	129	IgK	Homsap IGKV1-33*01 F	84.59	Homsap IGKJ4*02 F	89.47	CQIYTNESLTF
7	112	IgK	Homsap IGKV1-39*01 F	91.76	Homsap IGKJ4*01 F	97.37	CQQSNSTLSLTF
7	1	IgK	Homsap IGKV1-39*01 F	85.66	Homsap IGKJ4*01 F	89.47	CQQSNSSSSITF
7	126	IgK	Homsap IGKV1-39*01 F	87.1	Homsap IGKJ4*01 F	94.74	CQQSNSTLSLTF
7	101	IgK	Homsap IGKV1-5*01 F	93.55	Homsap IGKJ1*01 F	94.29	CQQYVSYSTF
7	167	IgK	Homsap IGKV1-9*01 F	98.57	Homsap IGKJ4*01 F	94.29	CQQLNSYPFF
7	73	IgL	Homsap IGLV1-51*01 F	90.88	Homsap IGLJ2*01 F	80.0	CGTWDSSLSVGLF
7	55	IgL	Homsap IGLV2-14*01 F	93.75	Homsap IGLJ2*01 F	100.0	CNAYASISPLF
7	11	IgL	Homsap IGLV6-57*01 F	90.38	Homsap IGLJ2*01 F	86.11	CQSFDSSGLIF
7	6	IgL	Homsap IGLV6-57*01 F	95.53	Homsap IGLJ2*01 F	83.33	CQSYDVSGLVF
7	10	IgL	Homsap IGLV6-57*01 F	90.03	Homsap IGLJ2*01 F	86.84	CQSYDASGLVF
7	12	IgL	Homsap IGLV6-57*01 F	89.69	Homsap IGLJ2*01 F	86.11	CQSFDSSGLIF
8	158	IgK	Homsap IGKV2-28*01 F	98.98	Homsap IGKJ4*01 F	97.22	CMQVLQTLTF
8	46	IgL	Homsap IGLV1-47*01 F	90.18	Homsap IGLJ3*02 F	100.0	CATWHDIDSWVF
9	127	IgK	Homsap IGKV1-9*01 F	97.85	Homsap IGKJ3*01 F	94.74	CQQLDSNPFTF
9	96	IgK	Homsap IGKV3-20*01 F	90.78	Homsap IGKJ2*04 F	97.37	CHHYGFSPCSF

Donor	Sequence	IgK/IgL	V-GENE and allele	V-REGION identity %	J-GENE and allele	J-REGION identity %	AA JUNCTION
Single cell-sorted ACPA-LC cells							
9	143	IgK	Homsap IGKV4-1*01 F	87.88	Homsap IGKJ3*01 F	89.47	CQQHYLPPFTF
9	147	IgK	Homsap IGKV4-1*01 F	94.61	Homsap IGKJ2*03 F	89.47	CQQYYITPYSF
9	146	IgK	Homsap IGKV4-1*01 F	95.29	Homsap IGKJ2*03 F	92.11	CQQYYITPYSF
9	84	IgK	Homsap IGKV4-1*01 F	81.14	Homsap IGKJ4*01 F	89.47	CLQYFSSELNF
9	171	IgL	Homsap IGLV3-10*01 F	93.19	Homsap IGLJ2*01 F	94.29	CYSSDTSGYQRVF
11	119	IgK	Homsap IGKV1-5*01 F	82.08	Homsap IGKJ4*01 F	76.32	CQQFDTYPLSF
11	151	IgK	Homsap IGKV1-5*03 F	88.17	Homsap IGKJ4*01 F	89.47	CQQFYSYPLTF
11	150	IgK	Homsap IGKV1-5*03 F	86.74	Homsap IGKJ1*01 F	92.11	CQQYNQYPWTF
11	162	IgK	Homsap IGKV2-28*01 F	91.16	Homsap IGKJ2*02 F	84.21	CMQDILIPCTF
11	155	IgK	Homsap IGKV2-28*01 F	85.03	Homsap IGKJ2*02 F	97.37	CMQDLMIPCTF
11	160	IgK	Homsap IGKV2-30*02 [F]	82.65	Homsap IGKJ5*01 F	94.74	CMQGSHWPPITF
11	161	IgK	Homsap IGKV2-30*02 [F]	82.65	Homsap IGKJ5*01 F	92.11	CMQGSHWPPIPF
11	130	IgK	Homsap IGKV4-1*01 F	87.54	Homsap IGKJ2*01 F	92.11	CQQYYEAPYTF
11	85	IgK	Homsap IGKV4-1*01 F	83.16	Homsap IGKJ2*01 F	86.84	CLQYLDAPYTF
11	148	IgK	Homsap IGKV4-1*01 F	82.15	Homsap IGKJ2*01 F	89.47	CQQYFRAPSNF
11	132	IgK	Homsap IGKV4-1*01 F	84.18	Homsap IGKJ2*01 F	84.21	CHQYFDPPYTF
11	2	IgK	Homsap IGKV4-1*01 F	74.75	Homsap IGKJ2*01 F	86.84	CFQYCCPPYTF
11	154	IgK	Homsap IGKV4-1*01 F	82.83	Homsap IGKJ2*03 F	86.84	CLQYLAAPYSF
11	142	IgK	Homsap IGKV4-1*01 F	79.46	Homsap IGKJ2*01 F	92.11	CLQYHAEPYTF
11	153	IgK	Homsap IGKV4-1*01 F	80.13	Homsap IGKJ2*01 F	84.21	CHQYLDPPYTF
11	23	IgL	Homsap IGLV1-44*01 F	79.3	Homsap IGLJ2*01 F	85.71	CASWDDSLEVVFF
11	58	IgL	Homsap IGLV1-47*01 F	87.72	Homsap IGLJ1*01 F	82.61	CATWDDSRSFVF
11	24	IgL	Homsap IGLV1-47*01 F	92.98	Homsap IGLJ1*01 F	86.84	CAAWDDNLKYVF
11	70	IgL	Homsap IGLV1-47*01 F	87.02	Homsap IGLJ3*02 F	81.58	CATWDDSLEESRWVF
11	74	IgL	Homsap IGLV1-47*01 F	83.86	Homsap IGLJ1*01 F	72.73	CAAWDDTPNFLF
11	48	IgL	Homsap IGLV1-47*01 F	71.99	Homsap IGLJ3*02 F	81.58	CATWDVGRGDDDWDWVF
11	47	IgL	Homsap IGLV1-47*02 F	74.82	Homsap IGLJ3*02 F	80.65	CATWDVSRDDRDWVF
11	76	IgL	Homsap IGLV1-47*02 F	82.11	Homsap IGLJ1*01 F	85.71	CATWDDTPTFLF
11	67	IgL	Homsap IGLV1-51*01 F	94.04	Homsap IGLJ2*01 F	97.22	CGTWDNSLSGVVF
11	51	IgL	Homsap IGLV2-11*01 F	87.13	Homsap IGLJ2*01 F	76.32	CSSYEGTFLF
11	170	IgL	Homsap IGLV3-25*03 F	80.65	Homsap IGLJ2*01 F	81.58	CQAADFSGHVVF
12	178	IgL	Homsap IGLV3-10*01 F	90.32	Homsap IGLJ3*02 F	79.31	CYSTDTDGKGVF
12	182	IgL	Homsap IGLV3-10*01 F	93.19	Homsap IGLJ2*01 F	73.68	CYSTDTSGNGVF
13	120	IgK	Homsap IGKV1-5*01 F	82.8	Homsap IGKJ4*01 F	76.32	CQKYKVF
13	21	IgL	Homsap IGLV1-44*01 F	82.81	Homsap IGLJ3*02 F	81.58	CASWDDRLRGWFF
13	49	IgL	Homsap IGLV1-44*01 F	82.11	Homsap IGLJ3*02 F	86.84	CAVWDNGLPGWVF
13	50	IgL	Homsap IGLV1-44*01 F	80.35	Homsap IGLJ3*02 F	84.21	CAVWDNGLPGWVF
13	66	IgL	Homsap IGLV1-44*01 F	80.0	Homsap IGLJ1*01 F	84.21	CGSWDNVLDGLYVF
13	28	IgL	Homsap IGLV4-69*01 F	95.92	Homsap IGLJ3*02 F	90.32	CQTWANGTWVF
14	110	IgK	Homsap IGKV1-33*01 F	95.7	Homsap IGKJ5*01 F	94.74	CQQYNIVPITF
14	134	IgK	Homsap IGKV4-1*01 F	71.72	Homsap IGKJ3*01 F	75.68	CQQYFSVPFTF
14	133	IgK	Homsap IGKV4-1*01 F	75.08	Homsap IGKJ3*01 F	78.95	CQQYLALPFTF
14	22	IgL	Homsap IGLV1-51*01 F	96.14	Homsap IGLJ3*02 F	94.29	CGTWDNSLSIGVF
14	68	IgL	Homsap IGLV1-51*01 F	85.26	Homsap IGLJ3*02 F	97.14	CATWDSGLNIGVF
14	69	IgL	Homsap IGLV1-51*01 F	87.72	Homsap IGLJ3*02 F	85.71	CGTWDDSLRAGVF
14	61	IgL	Homsap IGLV1-51*01 F	90.18	Homsap IGLJ2*01 F	89.29	CGAWDNSLRSGVF
14	77	IgL	Homsap IGLV2-23*02 F	78.12	Homsap IGLJ3*02 F	94.44	CCSYAGYSTWVF
14	191	IgL	Homsap IGLV3-21*02 F	74.55	Homsap IGLJ2*01 F	81.25	CHIWDNSVDRHVF
14	19	IgL	Homsap IGLV4-69*01 F	74.15	Homsap IGLJ2*01 F	74.19	CQTWGTHVELF

Donor	Sequence	IgK/IgL	V-GENE and allele	V-REGION identity %	J-GENE and allele	J-REGION identity %	AA JUNCTION
Single cell-sorted ACPA-LC cells							
15	118	IgK	Homsap IGKV1-39*01 F	78.14	Homsap IGKJ3*01 F	86.49	CQQSYMTPFTF
15	185	IgL	Homsap IGLV3-21*02 F	88.53	Homsap IGLJ3*02 F	86.84	CQVWERSGDYLVF
16	14	IgL	Homsap IGLV6-57*01 F	91.75	Homsap IGLJ2*01 F	83.78	CHSYDNTDLIF
17	113	IgK	Homsap IGKV1-33*01 F	97.85	Homsap IGKJ2*01 F	89.47	CQQYDFLPPVAF
17	98	IgK	Homsap IGKV2-28*01 F	94.22	Homsap IGKJ2*01 F	97.37	CMQVLQIPYTF
17	140	IgK	Homsap IGKV4-1*01 F	94.28	Homsap IGKJ3*01 F	92.11	CQQFYSTPFTF
17	187	IgL	Homsap IGLV3-27*01 F	95.34	Homsap IGLJ2*01 F	89.47	CYSAADDNSGVF
17	29	IgL	Homsap IGLV4-69*02 F	90.48	Homsap IGLJ2*01 F	86.49	CQTWGGGVVVF
17	5	IgL	Homsap IGLV6-57*02 F	95.88	Homsap IGLJ2*01 F	94.74	CQSYNDIPVF
17	7	IgL	Homsap IGLV6-57*02 F	90.72	Homsap IGLJ2*01 F	86.67	CQSFHNGDMIF
17	13	IgL	Homsap IGLV6-57*02 F	89.35	Homsap IGLJ2*01 F	91.89	CQSYDRGNMVF
17	8	IgL	Homsap IGLV6-57*02 F	91.07	Homsap IGLJ2*01 F	93.33	CQSYHRGNMVF
18	114	IgK	Homsap IGKV1-5*03 F	99.28	Homsap IGKJ2*04 F	100.0	CQQYNSYMCSF
18	169	IgK	Homsap IGKV1D-13*01 F	88.53	Homsap IGKJ5*01 F	94.74	CQQFSDYPSITF
18	82	IgK	Homsap IGKV3-15*01 F	100.0	Homsap IGKJ1*01 F	94.74	CQQYNNWPPGGTF
18	90	IgK	Homsap IGKV3-20*01 F	98.58	Homsap IGKJ1*01 F	100.0	CQQYGRSLTWTF
19	105	IgK	Homsap IGKV1-16*02 [F]	100.0	Homsap IGKJ4*01 F	100.0	CQQYNSYPLTF
19	111	IgK	Homsap IGKV1-39*01 F	99.64	Homsap IGKJ4*01 F	92.11	CQQSYSTPPGTF
19	88	IgK	Homsap IGKV2-28*01 F	93.54	Homsap IGKJ4*01 F	94.74	CMQTLKTFITF
19	87	IgK	Homsap IGKV2-28*01 F	94.56	Homsap IGKJ4*01 F	94.74	CMQTLETFITF
19	86	IgK	Homsap IGKV2D-29*01 F	91.84	Homsap IGKJ5*01 F	89.47	CVQSIRLPVTF
19	95	IgK	Homsap IGKV2D-29*01 F	91.16	Homsap IGKJ4*02 F	81.82	CVQSIRLPVTF
19	157	IgK	Homsap IGKV3-15*01 F	86.02	Homsap IGKJ2*01 F	81.58	CLQYDSWPYTF
19	83	IgK	Homsap IGKV3-15*01 F	100.0	Homsap IGKJ2*03 F	100.0	CQQYNNWPPYSF
19	92	IgK	Homsap IGKV3-20*01 F	88.65	Homsap IGKJ2*03 F	89.47	CQQYGDSPPYSF
19	131	IgK	Homsap IGKV4-1*01 F	87.41	Homsap IGKJ2*01 F	92.11	CQQYGIPPYTF
19	89	IgK	Homsap IGKV6-21*02 F	100.0	Homsap IGKJ1*01 F	97.37	CHQSSSLPGTF
19	60	IgL	Homsap IGLV1-40*01 F	91.32	Homsap IGLJ2*01 F	88.57	CQSYDINVTGSIF
19	57	IgL	Homsap IGLV1-47*01 F	92.28	Homsap IGLJ3*02 F	92.11	CASWDDGLGRPEWVF
19	64	IgL	Homsap IGLV1-47*01 F	100.0	Homsap IGLJ2*01 F	100.0	CAAWDDSLRGVF
19	190	IgL	Homsap IGLV3-1*01 F	79.21	Homsap IGLJ1*01 F	76.32	CQSWDTTTSLAFGTATTSLVF
19	189	IgL	Homsap IGLV3-1*01 F	79.57	Homsap IGLJ1*01 F	71.43	CQSWDTTTSLAFGTATTSLVF
19	180	IgL	Homsap IGLV3-1*01 F	83.15	Homsap IGLJ1*01 F	70.0	CQAWDITTYASGIMVNVPIVF
19	188	IgL	Homsap IGLV3-1*01 F	79.57	Homsap IGLJ1*01 F	75.0	CQSWDTTTSLAFGTATTSLVF
19	184	IgL	Homsap IGLV3-21*02 F	81.72	Homsap IGLJ2*01 F	87.5	CQAYDSVSEEVVF
19	39	IgL	Homsap IGLV4-69*01 F	100.0	Homsap IGLJ3*02 F	92.11	CQTWGTGIRGVF
20	128	IgK	Homsap IGKV1-27*01 F	93.55	Homsap IGKJ2*01 F	94.74	CQHYYTAPYTF
20	123	IgK	Homsap IGKV1-27*01 F	86.02	Homsap IGKJ2*01 F	86.84	CQQYDTAPYIF
20	122	IgK	Homsap IGKV1-27*01 F	88.53	Homsap IGKJ2*01 F	84.21	CQQYDTAPYIF
20	121	IgK	Homsap IGKV1-27*01 F	91.76	Homsap IGKJ2*01 F	89.47	CQKYDSVPYAF
20	172	IgK	Homsap IGKV1-27*01 F	90.32	Homsap IGKJ2*01 F	86.84	CQKYDSPPYNF
20	107	IgK	Homsap IGKV1-33*01 F	96.77	Homsap IGKJ4*01 F	92.11	CQLYDILPRLTF
20	106	IgK	Homsap IGKV1-33*01 F	97.13	Homsap IGKJ4*01 F	92.11	CQLYDILPRLTF
20	103	IgK	Homsap IGKV1-39*01 F	91.4	Homsap IGKJ2*01 F	94.74	CQQSYNPPYTF
20	102	IgK	Homsap IGKV1-39*01 F	95.34	Homsap IGKJ4*01 F	94.74	CQQSYSTLALTF
20	117	IgK	Homsap IGKV1-5*01 F	89.25	Homsap IGKJ1*01 F	92.11	CKQYYDLWTF
20	125	IgK	Homsap IGKV1-5*01 F	89.25	Homsap IGKJ1*01 F	91.67	CQHYDDYSRTF
20	115	IgK	Homsap IGKV1-5*01 F	92.83	Homsap IGKJ1*01 F	97.37	CQQYDDFPWTF
20	116	IgK	Homsap IGKV1-5*01 F	91.76	Homsap IGKJ1*01 F	94.44	CQHYDDYSRAF

Donor	Sequence	IgK/IgL	V-GENE and allele	V-REGION identity %	J-GENE and allele	J-REGION identity %	AA JUNCTION
Single cell-sorted ACPA-LC cells							
20	173	IgK	Homsap IGKV1-5*03 F	90.68	Homsap IGKJ3*01 F	97.37	CQQYGTESPIFTF
20	166	IgK	Homsap IGKV1-6*01 F	91.76	Homsap IGKJ2*01 F	97.37	CLQDHNFPNTF
20	175	IgK	Homsap IGKV1-6*01 F	87.1	Homsap IGKJ3*01 F	81.08	CLQDSSYPMIF
20	168	IgK	Homsap IGKV1-9*01 F	93.91	Homsap IGKJ4*01 F	94.74	CQHFNSYPLTF
20	159	IgK	Homsap IGKV2-28*01 F	88.78	Homsap IGKJ1*01 F	97.37	CMQWLQTPWTF
20	165	IgK	Homsap IGKV3-15*01 F	89.61	Homsap IGKJ2*01 F	97.37	CQQYHEWPYTF
20	91	IgK	Homsap IGKV3-20*01 F	93.62	Homsap IGKJ3*01 F	97.37	CQQYETSPLTF
20	139	IgK	Homsap IGKV4-1*01 F	94.95	Homsap IGKJ1*01 F	97.22	CQQYFETPRTF
20	137	IgK	Homsap IGKV4-1*01 F	91.67	Homsap IGKJ2*01 F	97.44	CQQYSTFYTF
20	141	IgK	Homsap IGKV4-1*01 F	90.97	Homsap IGKJ2*01 F	94.87	CQQYFTAYTF
20	62	IgL	Homsap IGLV1-40*01 F	93.06	Homsap IGLJ1*01 F	91.43	CQSFDSIFQYVF
20	56	IgL	Homsap IGLV1-40*01 F	94.79	Homsap IGLJ3*02 F	91.43	CQSYDNTLSGVF
20	65	IgL	Homsap IGLV1-44*01 F	88.77	Homsap IGLJ3*02 F	89.47	CAAWDYTLTGPTWLF
20	59	IgL	Homsap IGLV1-51*01 F	85.61	Homsap IGLJ1*01 F	97.37	CLTWDSDLNGYVF
20	177	IgL	Homsap IGLV3-10*01 F	90.32	Homsap IGLJ2*01 F	83.87	CYSTDSSGDRGVF
20	186	IgL	Homsap IGLV3-10*01 F	88.53	Homsap IGLJ2*01 F	86.84	CYTTDTIGDLGIF
20	179	IgL	Homsap IGLV3-25*03 F	93.19	Homsap IGLJ3*02 F	94.74	CQSTDSGGVWVF
20	75	IgL	Homsap IGLV3-25*03 F	84.95	Homsap IGLJ3*02 F	100.0	CQSVDTRGTWVF
20	44	IgL	Homsap IGLV4-69*01 F	92.52	Homsap IGLJ2*01 F	92.11	CQTWGTGLHVVF
20	42	IgL	Homsap IGLV4-69*01 F	92.86	Homsap IGLJ2*01 F	95.24	CQTWGTGLHVVF
20	43	IgL	Homsap IGLV4-69*01 F	92.86	Homsap IGLJ2*01 F	92.86	CQTWGTGLHVVF
20	40	IgL	Homsap IGLV4-69*01 F	95.92	Homsap IGLJ2*01 F	100.0	CQTWGTGLHVVF
20	45	IgL	Homsap IGLV4-69*01 F	93.2	Homsap IGLJ2*01 F	92.11	CQTWGTGFYVVF
20	26	IgL	Homsap IGLV5-37*01 F	99.02	Homsap IGLJ2*01 F	100.0	CMIWPSNAVVF
20	27	IgL	Homsap IGLV5-37*01 F	99.02	Homsap IGLJ2*01 F	100.0	CMIWPSNAVVF
20	9	IgL	Homsap IGLV6-57*01 F	94.5	Homsap IGLJ3*02 F	97.06	CQSYDDNNWVF
20	15	IgL	Homsap IGLV6-57*02 F	92.1	Homsap IGLJ3*02 F	89.47	CQSYDTETRVF
21	93	IgK	Homsap IGKV3-20*01 F	87.94	Homsap IGKJ5*01 F	92.11	CQQYAVSPVTF
21	94	IgK	Homsap IGKV3-20*01 F	87.94	Homsap IGKJ5*01 F	92.11	CQQYAVSPVTF
21	163	IgK	Homsap IGKV3-20*01 F	90.78	Homsap IGKJ2*01 F	87.18	CQQYGNSLYTF
21	138	IgK	Homsap IGKV4-1*01 F	96.3	Homsap IGKJ1*01 F	89.47	CQQFYSTPWAF
21	20	IgL	Homsap IGLV4-69*01 F	84.01	Homsap IGLJ7*01 F	84.21	CQTWGIDTYVVF
22	97	IgK	Homsap IGKV2-29*02 F	86.39	Homsap IGKJ2*01 F	89.47	CMQGIRLPFTF
22	164	IgK	Homsap IGKV4-1*01 F	86.87	Homsap IGKJ3*01 F	92.11	CQQHYTTPFTF
22	135	IgK	Homsap IGKV4-1*01 F	89.9	Homsap IGKJ1*01 F	96.88	CQPVGWTF
22	156	IgK	Homsap IGKV4-1*01 F	85.19	Homsap IGKJ1*01 F	93.75	CQSPGWTF
22	25	IgL	Homsap IGLV1-51*01 F	86.67	Homsap IGLJ1*01 F	92.11	CGAWDSGLSPGFVF
22	78	IgL	Homsap IGLV1-51*01 F	88.07	Homsap IGLJ1*01 F	92.11	CGAWDSSLGGGYVF
22	4	IgL	Homsap IGLV6-57*02 F	92.78	Homsap IGLJ3*02 F	94.74	CQSYDNGLWVL
23	72	IgL	Homsap IGLV1-47*01 F	82.46	Homsap IGLJ3*02 F	86.84	CAAWDAGLSGSWLF
23	176	IgL	Homsap IGLV3-25*02 F	86.02	Homsap IGLJ2*01 F	97.3	CQSGDTRTAYVVF
24	53	IgL	Homsap IGLV2-23*01 F	85.82	Homsap IGLJ2*01 F	85.71	CSSYVHPNKLTF
24	52	IgL	Homsap IGLV2-23*01 F	88.19	Homsap IGLJ2*01 F	85.71	CSSYVHRNKLTF
24	181	IgL	Homsap IGLV3-10*01 F	97.13	Homsap IGLJ2*01 F	89.47	CYSTDSSARGIF
Pool-sorted ACPA-LC clones							
1	K173	IgK	Homsap IGKV1-33*01 F	82.8	Homsap IGKJ4*01 F	86.84	CQQYRGLLSITF
1	K178	IgK	Homsap IGKV1-33*01 F	83.15	Homsap IGKJ2*02 F	77.78	CQQYLELARHF
1	K179	IgK	Homsap IGKV1-33*01 F	81.0	Homsap IGKJ4*01 F	86.11	CQQYFGLPLSF
1	K174	IgK	Homsap IGKV1-39*01 F	88.89	Homsap IGKJ3*01 F	94.59	CQQSHSVPFTF

Donor	Sequence	IgK/IgL	V-GENE and allele	V-REGION identity %	J-GENE and allele	J-REGION identity %	AA JUNCTION
Pool-sorted ACPA-LC clones							
1	K177	IgK	Homsap IGKV1-39*01 F	89.25	Homsap IGKJ3*01 F	94.59	CQQSHSVSFTF
1	K172	IgK	Homsap IGKV1-9*01 F	86.02	Homsap IGKJ1*01 F	91.43	CQQLSDFPSF
1	K176	IgK	Homsap IGKV3D-20*01 F	91.13	Homsap IGKJ5*01 F	89.47	CQQYANSPSTF
1	K175	IgK	Homsap IGKV4-1*01 F	88.89	Homsap IGKJ2*01 F	82.86	CQQYLEPPPTF
1	L109	IgL	Homsap IGLV1-40*01 F	90.24	Homsap IGLJ7*01 F	97.37	CQSYDRRLSGYAVF
1	L111	IgL	Homsap IGLV1-51*01 F	85.96	Homsap IGLJ1*01 F	84.21	CGTWDTSLEEEVF
1	L112	IgL	Homsap IGLV2-23*02 F	94.44	Homsap IGLJ2*01 F	94.74	CCSYAVKSTFDVVSSFYVVF
1	L110	IgL	Homsap IGLV3-10*01 F	94.27	Homsap IGLJ2*01 F	89.47	CYSTDSGGRGVF
3	L113	IgL	Homsap IGLV2-14*01 F	98.26	Homsap IGLJ1*01 F	100.0	CSSYTISTTHVF
4	K182	IgK	Homsap IGKV1-39*01 F	90.94	Homsap IGKJ1*01 F	60.53	CQQSYRLPPWTF
4	K165	IgK	Homsap IGKV2-28*01 F	99.66	Homsap IGKJ4*01 F	97.22	CMQALQTPLTF
4	K170	IgK	Homsap IGKV3-20*01 F	87.59	Homsap IGKJ4*01 F	94.59	CQHYTEPPFTF
4	K181	IgK	Homsap IGKV3-20*01 F	88.3	Homsap IGKJ4*01 F	86.49	CQQYVTSPFAF
4	K154	IgK	Homsap IGKV4-1*01 F	98.32	Homsap IGKJ2*01 F	97.22	CQQHYSIPHTF
4	K166	IgK	Homsap IGKV4-1*01 F	92.57	Homsap IGKJ2*01 F	100.0	CQQYYFTPYTF
4	K167	IgK	Homsap IGKV4-1*01 F	92.59	Homsap IGKJ2*01 F	97.37	CQQYHGLPYTF
4	K168	IgK	Homsap IGKV4-1*01 F	92.59	Homsap IGKJ2*01 F	71.05	CQQHHSIPYTF
4	K169	IgK	Homsap IGKV4-1*01 F	95.22	Homsap IGKJ2*01 F	69.44	CQQHYSLPHTF
4	K171	IgK	Homsap IGKV4-1*01 F	92.39	Homsap IGKJ2*01 F	71.05	CQQYHSLPYTF
4	K183	IgK	Homsap IGKV4-1*01 F	98.63	Homsap IGKJ4*01 F	63.16	CQQHYNILTL
4	K184	IgK	Homsap IGKV4-1*01 F	96.62	Homsap IGKJ4*01 F	81.58	CQQYSDTPESF
4	L114	IgL	Homsap IGLV2-14*01 F	98.59	Homsap IGLJ2*01 F	94.59	CCSYTSSSTLELF
4	L116	IgL	Homsap IGLV2-23*02 F	97.22	Homsap IGLJ2*01 F	94.59	CCSYGGSSTLELF
4	L117	IgL	Homsap IGLV2-23*02 F	96.84	Homsap IGLJ2*01 F	63.16	CCSYGGSSTSSLFW
4	L106	IgL	Homsap IGLV4-69*01 F	90.75	Homsap IGLJ3*02 F	97.22	CQTWDTGIRVF
4	L115	IgL	Homsap IGLV4-69*02 F	87.07	Homsap IGLJ3*02 F	91.67	CQTWDTDIRVF
5	K163	IgK	Homsap IGKV1-33*01 F	92.11	Homsap IGKJ5*01 F	94.74	CQEYDDILSIAF
5	K156	IgK	Homsap IGKV3-20*01 F	87.23	Homsap IGKJ2*01 F	89.47	CQQYGHSPTTF
5	K161	IgK	Homsap IGKV3-20*01 F	89.72	Homsap IGKJ2*01 F	92.11	CQQYGNSPSTF
5	K164	IgK	Homsap IGKV3D-20*01 F	86.64	Homsap IGKJ2*01 F	72.22	CQQYGNSLHF
5	L102	IgL	Homsap IGLV1-44*01 F	82.11	Homsap IGLJ3*02 F	77.14	CAAWDDTLGGLF
5	L100	IgL	Homsap IGLV1-47*01 F	94.31	Homsap IGLJ1*01 F	92.11	CASWDDSLSDRFVF
5	L103	IgL	Homsap IGLV1-47*01 F	85.92	Homsap IGLJ3*02 F	92.11	CATWNKANWEF
5	L99	IgL	Homsap IGLV4-69*01 F	87.76	Homsap IGLJ3*02 F	94.59	CQTWGAGVWVF
5	L105	IgL	Homsap IGLV4-69*01 F	86.21	Homsap IGLJ2*01 F	68.42	CQTWALRLGV
8	K128	IgK	Homsap IGKV1-33*01 F	91.04	Homsap IGKJ3*01 F	84.21	CQHYDKIGVIF
8	K152	IgK	Homsap IGKV1-33*01 F	82.8	Homsap IGKJ4*01 F	84.21	CQQYDDHPRVAF
8	K122	IgK	Homsap IGKV1-39*01 F	94.27	Homsap IGKJ5*01 F	89.47	CQQSFSTRAISF
8	K144	IgK	Homsap IGKV1-39*01 F	90.29	Homsap IGKJ5*01 F	94.74	CQQTYSSLTITF
8	K146	IgK	Homsap IGKV1-39*01 F	92.09	Homsap IGKJ5*01 F	86.84	CQQSYSTLAISF
8	K147	IgK	Homsap IGKV1-39*01 F	89.96	Homsap IGKJ2*01 F	92.11	CQHYYSRPPYNF
8	K148	IgK	Homsap IGKV1-39*01 F	94.89	Homsap IGKJ5*01 F	86.84	CQQSFSTRTISF
8	K151	IgK	Homsap IGKV1-39*01 F	92.09	Homsap IGKJ2*02 F	84.21	CQQSSSTLRVTF
8	K153	IgK	Homsap IGKV1-39*01 F	97.48	Homsap IGKJ2*01 F	94.74	CQQSSSTPHTF
8	K150	IgK	Homsap IGKV1-39*02 P	81.82	Homsap IGKJ5*01 F	94.74	VSRVTVPAITF
8	K145	IgK	Homsap IGKV3-15*01 F	92.34	Homsap IGKJ2*01 F	72.22	CQHYYSRPHTI
8	L95	IgL	Homsap IGLV1-47*01 F	86.97	Homsap IGLJ3*02 F	100.0	CAAWDKFMDGFWVF
8	L96	IgL	Homsap IGLV1-47*01 F	89.82	Homsap IGLJ3*02 F	94.74	CATWNTPDGFWVF

Donor	Sequence	IgK/IgL	V-GENE and allele	V-REGION identity %	J-GENE and allele	J-REGION identity %	AA JUNCTION
Pool-sorted ACPA-LC clones							
8	L50	IgL	Homsap IGLV3-21*01 F	90.65	Homsap IGLJ2*01 F	86.84	CQVWDNYSDHPVVF
8	L92	IgL	Homsap IGLV6-57*01 F	91.41	Homsap IGLJ2*01 F	86.84	CQSYDNITVVF
10	K142	IgK	Homsap IGKV1-39*01 F	85.97	Homsap IGKJ5*01 F	91.43	CQHSKSPFPTF
10	K140	IgK	Homsap IGKV1-39*01 F	79.93	Homsap IGKJ5*01 F	89.47	CHQTFSVISITF
10	K137	IgK	Homsap IGKV1-5*03 F	90.68	Homsap IGKJ2*01 F	84.62	CQQYSRPPYTF
10	K139	IgK	Homsap IGKV1-5*03 F	90.18	Homsap IGKJ2*01 F	67.65	CQQYSRPPTL
10	K141	IgK	Homsap IGKV1-NL1*01 F	85.14	Homsap IGKJ2*01 F	89.47	CQQYFSPPYTF
10	K136	IgK	Homsap IGKV2-40*01 F	88.89	Homsap IGKJ2*01 F	89.47	CMQRREFPSYTF
10	K138	IgK	Homsap IGKV4-1*01 F	82.94	Homsap IGKJ1*01 F	94.74	CQEYYGESRTF
11	K25	IgK	Homsap IGKV1-33*01 F	93.91	Homsap IGKJ4*01 F	97.22	CQQYDGVPLTF
11	K53	IgK	Homsap IGKV1-33*01 F	85.55	Homsap IGKJ4*01 F	78.95	CQHYDDLPVACSLNIFHHISVNTIVVFHF
11	K92	IgK	Homsap IGKV1-33*01 F	93.19	Homsap IGKJ4*01 F	97.22	CQQYDTVPLTF
11	K72	IgK	Homsap IGKV1-33*01 F	82.93	Homsap IGKJ4*01 F	63.16	CQHMMFPLTF
11	K26	IgK	Homsap IGKV1-39*01 F	88.17	Homsap IGKJ4*01 F	89.47	CQQSFSTVALTF
11	K68	IgK	Homsap IGKV1-39*01 F	86.74	Homsap IGKJ4*01 F	81.08	CQQGYTTPGNF
11	K91	IgK	Homsap IGKV1-39*01 F	96.06	Homsap IGKJ2*03 F	97.44	CQQSYSTRYSF
11	K100	IgK	Homsap IGKV1-39*01 F	96.77	Homsap IGKJ1*01 F	100.0	CQQYNSYPTF
11	K48	IgK	Homsap IGKV1-39*01 F	82.73	Homsap IGKJ4*01 F	81.08	CQQTLTIPGNF
11	K64	IgK	Homsap IGKV1-39*01 F	84.23	Homsap IGKJ1*01 F	72.97	CQQGLTIPGNF
11	K71	IgK	Homsap IGKV1-39*01 F	83.51	Homsap IGKJ1*01 F	70.27	CQRGLAIPGNF
11	K125	IgK	Homsap IGKV1-39*01 F	84.23	Homsap IGKJ1*01 F	72.97	CQQGLTVPGNF
11	K34	IgK	Homsap IGKV1-5*01 F	84.84	Homsap IGKJ4*01 F	78.95	CQQFDTYPLSF
11	K35	IgK	Homsap IGKV1-5*03 F	88.17	Homsap IGKJ4*01 F	84.21	CQQYDVYPLAF
11	K74	IgK	Homsap IGKV1-5*03 F	100.0	Homsap IGKJ1*01 F	94.59	CQQYNSYPTF
11	K120	IgK	Homsap IGKV1-5*03 F	83.87	Homsap IGKJ4*01 F	89.47	CQQFLAFPLTF
11	K29	IgK	Homsap IGKV1D-13*01 F	85.66	Homsap IGKJ3*01 F	81.58	CQHFRNNWFTF
11	K42	IgK	Homsap IGKV2-24*01 F	87.76	Homsap IGKJ1*01 F	89.47	CMQATHRPWTF
11	K98	IgK	Homsap IGKV2-29*01 P	87.76	Homsap IGKJ2*01 F	88.57	CMQGKDLLF
11	K1	IgK	Homsap IGKV2-30*02 [F]	94.86	Homsap IGKJ5*01 F	100.0	CMQATHWPPITF
11	K133	IgK	Homsap IGKV2-30*02 [F]	94.56	Homsap IGKJ5*01 F	100.0	CMQATHWSPITF
11	K134	IgK	Homsap IGKV2-30*02 [F]	92.98	Homsap IGKJ5*01 F	68.42	CMQATHCSDHL
11	K61	IgK	Homsap IGKV2-30*02 [F]	83.67	Homsap IGKJ5*01 F	89.47	CMQSSHWPPLTF
11	K56	IgK	Homsap IGKV3-15*01 F	89.21	Homsap IGKJ4*01 F	88.24	CQQYFEWASF
11	K87	IgK	Homsap IGKV3-15*01 F	89.25	Homsap IGKJ4*01 F	90.62	CQQYFEWAPF
11	K93	IgK	Homsap IGKV3-15*01 F	88.89	Homsap IGKJ1*01 F	94.74	CQHYNDWPWTF
11	K33	IgK	Homsap IGKV3-15*01 F	83.03	Homsap IGKJ2*01 F	84.21	CQQYHGNPPYNF
11	K59	IgK	Homsap IGKV3-15*01 F	83.81	Homsap IGKJ2*01 F	89.47	CQQYDRNPPYNF
11	K104	IgK	Homsap IGKV3-15*01 F	82.01	Homsap IGKJ2*01 F	89.47	CQHYDGNPPYNF
11	K23	IgK	Homsap IGKV3-20*01 F	92.91	Homsap IGKJ1*01 F	89.19	CQQYGNSPTTF
11	K51	IgK	Homsap IGKV3-20*01 F	88.26	Homsap IGKJ3*01 F	82.86	CQDYGTSPSF
11	K116	IgK	Homsap IGKV3-20*01 F	86.12	Homsap IGKJ1*01 F	81.82	CQEYKTGLF
11	K129	IgK	Homsap IGKV3-20*01 F	88.3	Homsap IGKJ3*01 F	84.38	CQDYGTSPPF
11	K135	IgK	Homsap IGKV3-20*01 F	87.86	Homsap IGKJ2*01 F	64.1	CQDYGLHPL
11	K63	IgK	Homsap IGKV3-20*02 F	85.61	Homsap IGKJ1*01 F	76.32	CQQYKTGLF
11	K70	IgK	Homsap IGKV3D-15*01 F	84.84	Homsap IGKJ1*01 F	81.82	CQQYKTGLF
11	K111	IgK	Homsap IGKV3D-20*02 ORF	81.43	Homsap IGKJ1*01 F	84.21	CQQYEKGTF
11	K10	IgK	Homsap IGKV4-1*01 F	92.93	Homsap IGKJ4*01 F	92.11	CQQYYSHVSLTF
11	K13	IgK	Homsap IGKV4-1*01 F	88.18	Homsap IGKJ4*01 F	97.37	CQQYLSSISLTF
11	K14	IgK	Homsap IGKV4-1*01 F	88.22	Homsap IGKJ2*01 F	92.11	CQQYYEAPYTF

Donor	Sequence	IgK/IgL	V-GENE and allele	V-REGION identity %	J-GENE and allele	J-REGION identity %	AA JUNCTION
Pool-sorted ACPA-LC clones							
11	K15	IgK	Homsap IGKV4-1*01 F	92.93	Homsap IGKJ4*01 F	86.84	CQQYYSELSLTF
11	K17	IgK	Homsap IGKV4-1*01 F	92.59	Homsap IGKJ1*01 F	86.84	CHQYYETPQMF
11	K20	IgK	Homsap IGKV4-1*01 F	90.24	Homsap IGKJ2*03 F	97.37	CLQYFDEPYSF
11	K27	IgK	Homsap IGKV4-1*01 F	93.2	Homsap IGKJ4*01 F	89.47	CQQYYSKLSLTF
11	K38	IgK	Homsap IGKV4-1*01 F	91.25	Homsap IGKJ4*01 F	94.74	CQQYYSFVSLTF
11	K39	IgK	Homsap IGKV4-1*01 F	91.92	Homsap IGKJ2*01 F	86.84	CQQYYGNPPYTF
11	K41	IgK	Homsap IGKV4-1*01 F	92.59	Homsap IGKJ4*01 F	89.47	CQQYYSSISMTF
11	K44	IgK	Homsap IGKV4-1*01 F	92.59	Homsap IGKJ4*01 F	94.74	CQQYYSTVSLTF
11	K54	IgK	Homsap IGKV4-1*01 F	92.26	Homsap IGKJ4*01 F	89.47	CQQYKSSISMTF
11	K62	IgK	Homsap IGKV4-1*01 F	91.92	Homsap IGKJ4*01 F	89.47	CQQYYSYISMTF
11	K65	IgK	Homsap IGKV4-1*01 F	93.81	Homsap IGKJ4*01 F	73.68	CQQYYSTFAHF
11	K66	IgK	Homsap IGKV4-1*01 F	91.89	Homsap IGKJ4*01 F	89.47	CQQYSSSISMTF
11	K67	IgK	Homsap IGKV4-1*01 F	94.28	Homsap IGKJ4*01 F	100.0	CQQYCSTISLTF
11	K73	IgK	Homsap IGKV4-1*01 F	90.57	Homsap IGKJ4*01 F	92.11	CQQYHTTPVSF
11	K76	IgK	Homsap IGKV4-1*01 F	90.91	Homsap IGKJ4*01 F	92.11	CQQYYTTPVSF
11	K79	IgK	Homsap IGKV4-1*01 F	91.03	Homsap IGKJ2*01 F	68.57	CQQYHTTPSV
11	K80	IgK	Homsap IGKV4-1*01 F	92.26	Homsap IGKJ4*01 F	94.74	CQQYYSSVSLTF
11	K82	IgK	Homsap IGKV4-1*01 F	93.94	Homsap IGKJ4*01 F	92.11	CQQYYSKVSLTF
11	K83	IgK	Homsap IGKV4-1*01 F	91.86	Homsap IGKJ2*02 F	72.22	CQQYYSRFRSL
11	K84	IgK	Homsap IGKV4-1*01 F	92.2	Homsap IGKJ2*02 F	63.89	CQQYYSSVRSL
11	K96	IgK	Homsap IGKV4-1*01 F	94.28	Homsap IGKJ4*01 F	97.37	CQQYCSTISFTF
11	K102	IgK	Homsap IGKV4-1*01 F	90.57	Homsap IGKJ4*01 F	94.74	CQQFYSLVSLTF
11	K105	IgK	Homsap IGKV4-1*01 F	89.86	Homsap IGKJ2*03 F	100.0	CLQYSSEPYSF
11	K106	IgK	Homsap IGKV4-1*01 F	85.19	Homsap IGKJ2*01 F	89.47	CLQYLNPPYTF
11	K109	IgK	Homsap IGKV4-1*01 F	92.59	Homsap IGKJ4*01 F	92.11	CQQYYSSVSITF
11	K112	IgK	Homsap IGKV4-1*01 F	92.54	Homsap IGKJ2*01 F	71.79	CHQYYVLFDHF
11	K114	IgK	Homsap IGKV4-1*01 F	87.76	Homsap IGKJ2*02 F	60.53	CLQYLMSRTV
11	K115	IgK	Homsap IGKV4-1*01 F	91.19	Homsap IGKJ4*01 F	89.47	CQQYYSFISMTF
11	K117	IgK	Homsap IGKV4-1*01 F	93.24	Homsap IGKJ2*01 F	68.57	CQQYYSSFPHF
11	K118	IgK	Homsap IGKV4-1*01 F	90.44	Homsap IGKJ4*01 F	68.57	CQQYFRLPSI
11	K119	IgK	Homsap IGKV4-1*01 F	91.58	Homsap IGKJ5*01 F	91.43	CQQYYSSPLF
11	K126	IgK	Homsap IGKV4-1*01 F	92.93	Homsap IGKJ4*01 F	92.11	CQQYYSTVSPTF
11	K127	IgK	Homsap IGKV4-1*01 F	91.58	Homsap IGKJ4*01 F	94.74	CQQYLCTVSLTF
11	K130	IgK	Homsap IGKV4-1*01 F	92.26	Homsap IGKJ4*01 F	92.11	CQQYYSWLSLTF
11	K12	IgK	Homsap IGKV4-1*01 F	81.82	Homsap IGKJ2*01 F	86.84	CQQYLRAPSNF
11	K16	IgK	Homsap IGKV4-1*01 F	84.85	Homsap IGKJ2*03 F	78.95	CLQYLDPPYSF
11	K18	IgK	Homsap IGKV4-1*01 F	82.43	Homsap IGKJ2*01 F	81.58	CHQYLDPPYTF
11	K24	IgK	Homsap IGKV4-1*01 F	78.79	Homsap IGKJ2*01 F	78.95	CLQYFVEPYTF
11	K30	IgK	Homsap IGKV4-1*01 F	78.11	Homsap IGKJ4*01 F	84.21	CQQYHATPVAF
11	K32	IgK	Homsap IGKV4-1*01 F	79.46	Homsap IGKJ2*01 F	92.11	CLQYHAEPYTF
11	K43	IgK	Homsap IGKV4-1*01 F	82.19	Homsap IGKJ2*01 F	68.75	CQQYLRLRPI
11	K45	IgK	Homsap IGKV4-1*01 F	84.35	Homsap IGKJ2*01 F	89.47	CHQYYAVPYNF
11	K46	IgK	Homsap IGKV4-1*01 F	79.12	Homsap IGKJ2*02 F	84.21	CQQYLRAPCIF
11	K47	IgK	Homsap IGKV4-1*01 F	83.5	Homsap IGKJ2*03 F	86.84	CLQYMDVPYSF
11	K49	IgK	Homsap IGKV4-1*01 F	83.27	Homsap IGKJ3*01 F	64.29	CLQYWIPYSF
11	K50	IgK	Homsap IGKV4-1*01 F	84.59	Homsap IGKJ1*01 F	61.76	CLQYLDPPTV
11	K52	IgK	Homsap IGKV4-1*01 F	78.79	Homsap IGKJ2*03 F	86.84	CQQYRRSPASF
11	K55	IgK	Homsap IGKV4-1*01 F	73.68	Homsap IGKJ2*01 F	61.76	CHQYLIRPIL
11	K57	IgK	Homsap IGKV4-1*01 F	82.83	Homsap IGKJ2*01 F	94.74	CLQYCCIPYTF

Donor	Sequence	IgK/IgL	V-GENE and allele	V-REGION identity %	J-GENE and allele	J-REGION identity %	AA JUNCTION
Pool-sorted ACPA-LC clones							
11	K58	IgK	Homsap IGKV4-1*01 F	82.15	Homsap IGKJ2*01 F	89.47	CQQYFRAPSNF
11	K69	IgK	Homsap IGKV4-1*01 F	79.04	Homsap IGKJ2*01 F	71.05	CHQYFEAPYMF
11	K75	IgK	Homsap IGKV4-1*01 F	77.44	Homsap IGKJ2*01 F	76.32	CLQYFAEPYSF
11	K77	IgK	Homsap IGKV4-1*01 F	78.38	Homsap IGKJ4*01 F	84.21	CQQYHATSVAF
11	K85	IgK	Homsap IGKV4-1*01 F	80.13	Homsap IGKJ2*01 F	86.84	CQQYIDVPFNF
11	K86	IgK	Homsap IGKV4-1*01 F	80.47	Homsap IGKJ2*01 F	89.19	CQQYIESFNF
11	K88	IgK	Homsap IGKV4-1*01 F	82.77	Homsap IGKJ2*03 F	92.11	CLQHYSEPYSF
11	K90	IgK	Homsap IGKV4-1*01 F	78.64	Homsap IGKJ2*02 F	63.16	CQQYRRSRGQF
11	K94	IgK	Homsap IGKV4-1*01 F	83.46	Homsap IGKJ2*02 F	66.67	CQQYCSSFRSL
11	K97	IgK	Homsap IGKV4-1*01 F	84.51	Homsap IGKJ4*01 F	86.84	CQQFHKTPVTF
11	K99	IgK	Homsap IGKV4-1*01 F	79.58	Homsap IGKJ4*01 F	63.16	CLQYCCIHTL
11	K101	IgK	Homsap IGKV4-1*01 F	84.85	Homsap IGKJ2*01 F	89.47	CLQYLDPPYTF
11	K103	IgK	Homsap IGKV4-1*01 F	73.74	Homsap IGKJ2*01 F	86.84	CFQYCCPPYTF
11	K107	IgK	Homsap IGKV4-1*01 F	82.49	Homsap IGKJ2*01 F	84.21	CQQYVVEPFDF
11	K108	IgK	Homsap IGKV4-1*01 F	74.66	Homsap IGKJ4*01 F	65.79	CFQYCCPLHF
11	K110	IgK	Homsap IGKV4-1*01 F	82.15	Homsap IGKJ2*01 F	86.84	CQQYFRAPSDF
11	K113	IgK	Homsap IGKV4-1*01 F	84.43	Homsap IGKJ2*01 F	74.36	CQQYFRFVQF
11	K121	IgK	Homsap IGKV4-1*01 F	85.66	Homsap IGKJ2*01 F	81.82	CQQYLELPQF
11	K124	IgK	Homsap IGKV4-1*01 F	78.45	Homsap IGKJ4*01 F	84.21	CQQYHARPVAF
11	K131	IgK	Homsap IGKV4-1*01 F	82.77	Homsap IGKJ2*03 F	89.74	CLQHYSEQYSF
11	L13	IgL	Homsap IGLV1-44*01 F	82.81	Homsap IGLJ2*01 F	78.95	CATWDDILHVVSF
11	L21	IgL	Homsap IGLV1-44*01 F	79.3	Homsap IGLJ2*01 F	78.95	CATWDDSLQVVSF
11	L23	IgL	Homsap IGLV1-44*01 F	62.86	Homsap IGLJ2*01 F	81.58	CASWDDSLEVVFF
11	L30	IgL	Homsap IGLV1-44*01 F	76.95	Homsap IGLJ5*02 ORF	68.42	CASWDDSWKLSF
11	L39	IgL	Homsap IGLV1-44*01 F	77.42	Homsap IGLJ2*01 F	63.16	CASWDDSLEVVF
11	L73	IgL	Homsap IGLV1-44*01 F	79.3	Homsap IGLJ2*01 F	76.32	CATWDDSLQVISF
11	L15	IgL	Homsap IGLV1-44*01 F	65.58	Homsap IGLJ2*01 F	78.95	YYCATWDDILHVVSF
11	L24	IgL	Homsap IGLV1-47*01 F	89.12	Homsap IGLJ1*01 F	84.21	CAIWDDNLKYVF
11	L37	IgL	Homsap IGLV1-47*01 F	89.47	Homsap IGLJ1*01 F	81.58	CAAWDATLRFVF
11	L54	IgL	Homsap IGLV1-47*01 F	91.2	Homsap IGLJ1*01 F	86.84	CAAWDDSLKYVF
11	L76	IgL	Homsap IGLV1-47*01 F	89.82	Homsap IGLJ1*01 F	86.84	CATWNDIPNFVF
11	L79	IgL	Homsap IGLV1-47*01 F	88.73	Homsap IGLJ1*01 F	81.08	CATWDDSRSFVF
11	L69	IgL	Homsap IGLV1-47*01 F	77.89	Homsap IGLJ3*02 F	76.32	CAAWDGDLRIWMF
11	L9	IgL	Homsap IGLV1-47*01 F	88.42	Homsap IGLJ3*02 F	89.47	CATWDRTYESNWLF
11	L22	IgL	Homsap IGLV1-47*01 F	88.42	Homsap IGLJ3*02 F	86.84	CATWDRTYESNWQF
11	L68	IgL	Homsap IGLV1-47*01 F	92.28	Homsap IGLJ1*01 F	84.21	CAAWHDSLRYVF
11	L72	IgL	Homsap IGLV1-47*01 F	92.63	Homsap IGLJ1*01 F	84.21	CAVWDDSLRYVF
11	L75	IgL	Homsap IGLV1-47*01 F	89.36	Homsap IGLJ3*02 F	94.74	CATWDRTYESNWVF
11	L80	IgL	Homsap IGLV1-47*01 F	91.23	Homsap IGLJ1*01 F	84.21	CAAWDDSLRYVF
11	L11	IgL	Homsap IGLV1-47*01 F	83.1	Homsap IGLJ3*02 F	86.84	CATWDHSFADSNWLF
11	L44	IgL	Homsap IGLV1-47*01 F	82.46	Homsap IGLJ3*02 F	86.84	CATWHDTLDDDIWVF
11	L46	IgL	Homsap IGLV1-47*01 F	84.21	Homsap IGLJ1*01 F	78.95	CAGWHDTLKFVF
11	L53	IgL	Homsap IGLV1-47*01 F	81.75	Homsap IGLJ3*02 F	84.21	CATWDHSFDDSNWLF
11	L63	IgL	Homsap IGLV1-47*01 F	83.51	Homsap IGLJ1*01 F	78.95	CTAWDEVLEFVF
11	L64	IgL	Homsap IGLV1-47*01 F	84.91	Homsap IGLJ1*01 F	66.67	CAAWDDSAQFLF
11	L70	IgL	Homsap IGLV1-47*01 F	82.59	Homsap IGLJ1*01 F	81.58	CAAWHKDVEYVF
11	L71	IgL	Homsap IGLV1-47*01 F	84.91	Homsap IGLJ3*02 F	86.84	CASWDDSFGGSSWIF
11	L58	IgL	Homsap IGLV1-47*02 F	87.02	Homsap IGLJ1*01 F	81.82	CATWDDSPQFLF
11	L10	IgL	Homsap IGLV1-47*02 F	82.75	Homsap IGLJ1*01 F	78.95	CATWDDKSTFFF

Donor	Sequence	IgK/IgL	V-GENE and allele	V-REGION identity %	J-GENE and allele	J-REGION identity %	AA JUNCTION
Pool-sorted ACPA-LC clones							
11	L26	IgL	Homsap IGLV1-47*02 F	81.75	Homsap IGLJ1*01 F	73.68	CATWDDTPTFVF
11	L28	IgL	Homsap IGLV1-47*02 F	81.75	Homsap IGLJ1*01 F	73.68	CATWDDTPIFVF
11	L29	IgL	Homsap IGLV1-47*02 F	81.4	Homsap IGLJ1*01 F	73.68	CATWGDTPTFVF
11	L77	IgL	Homsap IGLV1-47*02 F	80.7	Homsap IGLJ1*01 F	73.68	CATWDDTPTFFF
11	L40	IgL	Homsap IGLV1-51*01 F	100.0	Homsap IGLJ1*01 F	89.47	CGTWDSSLSAGGVF
11	L61	IgL	Homsap IGLV1-51*01 F	89.82	Homsap IGLJ2*01 F	94.44	CAIWDDTLSAVVF
11	L25	IgL	Homsap IGLV1-51*01 F	83.16	Homsap IGLJ2*01 F	71.05	CATWDSDLRAIFF
11	L17	IgL	Homsap IGLV2-11*01 F	88.19	Homsap IGLJ2*01 F	80.0	CCSYVGRDNFFF
11	L31	IgL	Homsap IGLV2-11*01 F	86.76	Homsap IGLJ2*01 F	78.95	CCAYLGEFLF
11	L32	IgL	Homsap IGLV2-11*01 F	92.01	Homsap IGLJ2*01 F	85.71	CCSFAGRDNFVF
11	L33	IgL	Homsap IGLV2-11*01 F	88.07	Homsap IGLJ2*01 F	68.75	CCSYVGRDNFF
11	L43	IgL	Homsap IGLV2-11*01 F	85.11	Homsap IGLJ2*01 F	78.95	CCIHIGFFIF
11	L57	IgL	Homsap IGLV2-11*01 F	88.19	Homsap IGLJ1*01 F	86.49	CYSYAGMFIFYVF
11	L65	IgL	Homsap IGLV2-11*01 F	87.23	Homsap IGLJ3*02 F	81.58	CCVHIGFFMF
11	L82	IgL	Homsap IGLV2-11*01 F	88.19	Homsap IGLJ1*01 F	86.49	CYSYAGMLIFYVF
11	L83	IgL	Homsap IGLV2-11*01 F	89.93	Homsap IGLJ2*01 F	85.71	CCSFAGKDNFVF
11	L85	IgL	Homsap IGLV2-11*01 F	90.97	Homsap IGLJ2*01 F	85.71	CCSFAGSDNFVF
11	L8	IgL	Homsap IGLV2-11*01 F	87.13	Homsap IGLJ2*01 F	76.32	CSSYEGTFLF
11	L34	IgL	Homsap IGLV2-11*01 F	76.92	Homsap IGLJ2*01 F	78.95	CSLYVGEFLF
11	L49	IgL	Homsap IGLV2-11*01 F	84.75	Homsap IGLJ1*01 F	76.32	CCIHIGFFIF
11	L51	IgL	Homsap IGLV2-11*01 F	84.25	Homsap IGLJ2*01 F	78.95	CSSYETFFLF
11	L52	IgL	Homsap IGLV2-11*01 F	84.62	Homsap IGLJ2*01 F	78.95	CSSYENFFLF
11	L81	IgL	Homsap IGLV2-11*01 F	73.19	Homsap IGLJ2*01 F	76.32	CSLYVGVFLF
11	L42	IgL	Homsap IGLV2-11*01 F	85.35	Homsap IGLJ2*01 F	81.58	CCSYVGYFIF
11	L12	IgL	Homsap IGLV2-11*01 F	81.88	Homsap IGLJ5*01 ORF	76.32	CCSYRGISEGNFVF
11	L89	IgL	Homsap IGLV2-11*01 F	75.0	Homsap IGLJ2*01 F	78.95	CCLYFGTFIF
11	L48	IgL	Homsap IGLV2-8*03 F	51.17	Homsap IGLJ2*01 F	63.16	CQQYHATPVAF
11	L86	IgL	Homsap IGLV3-16*01 F	96.06	Homsap IGLJ2*01 F	88.89	CLSADSSGPYHVF
11	L7	IgL	Homsap IGLV3-21*02 F	89.96	Homsap IGLJ2*01 F	84.21	CQVWNTITDQFVF
11	L78	IgL	Homsap IGLV3-21*02 F	88.53	Homsap IGLJ2*01 F	84.21	CQVWNFSSDQFVF
11	L18	IgL	Homsap IGLV3-25*03 F	90.68	Homsap IGLJ3*02 F	94.74	CQSADSSGLSWVF
11	L19	IgL	Homsap IGLV3-25*03 F	87.36	Homsap IGLJ2*01 F	78.95	CQSVDRTNRIIF
11	L20	IgL	Homsap IGLV3-25*03 F	89.93	Homsap IGLJ2*01 F	84.21	CQSVDRNNIIIF
11	L27	IgL	Homsap IGLV3-25*03 F	87.32	Homsap IGLJ2*01 F	84.21	CQSVNKKNIIIF
11	L35	IgL	Homsap IGLV3-25*03 F	89.57	Homsap IGLJ2*01 F	81.58	CQSVDSKNIIIF
11	L36	IgL	Homsap IGLV3-25*03 F	85.19	Homsap IGLJ1*01 F	68.42	CQSVTEEYYHF
11	L45	IgL	Homsap IGLV3-25*03 F	86.74	Homsap IGLJ2*01 F	84.21	CQTGNRSGTLIF
11	L74	IgL	Homsap IGLV3-25*03 F	90.68	Homsap IGLJ3*02 F	89.47	CQSADSSDFPVF
11	L88	IgL	Homsap IGLV3-25*03 F	84.53	Homsap IGLJ3*02 F	94.74	CQSADHSGTSWVF
11	L14	IgL	Homsap IGLV3-27*01 F	88.17	Homsap IGLJ1*01 F	71.05	CYSVTGWLGLF
11	L47	IgL	Homsap IGLV3-27*01 F	82.44	Homsap IGLJ1*01 F	76.32	CYSASGYIGVF
11	L56	IgL	Homsap IGLV6-57*01 F	83.04	Homsap IGLJ2*01 F	86.49	CESYNDTNIIF
11	L87	IgL	Homsap IGLV7-46*01 F	97.92	Homsap IGLJ2*01 F	100.0	CLLSYSAARVF
12	K8	IgK	Homsap IGKV1-17*01 F	100.0	Homsap IGKJ2*01 F	97.44	CLQHNSYSYTF
12	K3	IgK	Homsap IGKV3-20*01 F	92.2	Homsap IGKJ3*01 F	86.84	CQQYGTSPYTF
12	K5	IgK	Homsap IGKV3-20*01 F	90.39	Homsap IGKJ2*03 F	81.58	CQQYGEEPPYSF
12	K7	IgK	Homsap IGKV3-20*01 F	87.94	Homsap IGKJ2*01 F	89.47	CQQYGEESPYNF
10 & 11	L38	IgL	Homsap IGLV3-21*02 F	88.17	Homsap IGLJ2*01 F	97.22	CQGWDSGDNLLVF
11 & 12	K4	IgK	Homsap IGKV3-20*01 F	88.65	Homsap IGKJ2*02 F	81.58	CQLYEDSGFVF

Donor	Sequence	IgK/IgL	V-GENE and allele	V-REGION identity %	J-GENE and allele	J-REGION identity %	AA JUNCTION
Pool-sorted ACPA-LC clones							
11 & 12	K6	IgK	Homsap IGKV3-20*01 F	87.54	Homsap IGKJ2*01 F	89.47	CQQYGEEPPYNF
11 & 12	K2	IgK	Homsap IGKV4-1*01 F	91.25	Homsap IGKJ4*01 F	86.84	CQQYYSRLSLTF
11 & 12	L1	IgL	Homsap IGLV1-44*01 F	83.33	Homsap IGLJ2*01 F	83.78	CASWDGSLNGVLF
11 & 12	L3	IgL	Homsap IGLV1-47*01 F	80.0	Homsap IGLJ3*02 F	84.21	CAAWDGGLRTWLF
11 & 12	L2	IgL	Homsap IGLV3-25*03 F	86.74	Homsap IGLJ2*01 F	89.47	CQSADSTGFSMVF
11 & 12	L6	IgL	Homsap IGLV3-25*03 F	90.32	Homsap IGLJ2*01 F	84.21	CQSVDSNNIIIF
11 & 12	L5	IgL	Homsap IGLV3-25*03 F	83.15	Homsap IGLJ2*01 F	86.84	CQAADFSGHVVF
11 & 12	L4	IgL	Homsap IGLV6-57*02 F	80.41	Homsap IGLJ3*02 F	91.89	CHSYDDDNWVF
3 & 4	K180	IgK	Homsap IGKV4-1*01 F	93.71	Homsap IGKJ4*01 F	100.0	CQQYYNTPALTF
3 & 5	K159	IgK	Homsap IGKV2-28*01 F	80.95	Homsap IGKJ4*02 F	89.47	CMQSLLVFLSF
4 & 5	L107	IgL	Homsap IGLV2-14*02 F	92.01	Homsap IGLJ1*01 F	100.0	CTSYAGNVTVF
4 & 5	L98	IgL	Homsap IGLV2-23*02 F	95.49	Homsap IGLJ2*01 F	89.19	CCSYAGSSTLEVF
4 & 5	L97	IgL	Homsap IGLV2-8*01 F	91.67	Homsap IGLJ1*01 F	100.0	CTSYAGNVTVF
4 & 8	K143	IgK	Homsap IGKV3-20*01 F	87.94	Homsap IGKJ4*01 F	94.59	CQHYAEPPFTF
5 & 11	K31	IgK	Homsap IGKV1-33*01 F	91.76	Homsap IGKJ5*01 F	94.74	CQEYGDILSIAF
5 & 11	K37	IgK	Homsap IGKV1-39*01 F	93.55	Homsap IGKJ4*01 F	97.37	CQQSYSSLALTF
5 & 11	K9	IgK	Homsap IGKV1-5*03 F	83.87	Homsap IGKJ1*01 F	81.08	CQQYQKYATF
5 & 11	K40	IgK	Homsap IGKV2D-29*01 F	78.35	Homsap IGKJ4*01 F	94.44	CLQSVELPLTF
5 & 11	K22	IgK	Homsap IGKV3-20*01 F	84.34	Homsap IGKJ1*01 F	81.82	CQDYKTGLF
5 & 11	K162	IgK	Homsap IGKV3-20*01 F	88.97	Homsap IGKJ4*01 F	94.59	CQQYGNSPTTF
5 & 11	K155	IgK	Homsap IGKV3D-20*01 F	86.88	Homsap IGKJ4*01 F	94.59	CQQYGNSPTTF
5 & 11	K11	IgK	Homsap IGKV4-1*01 F	85.19	Homsap IGKJ2*02 F	94.44	CQQYYNMPRTF
5 & 11	K21	IgK	Homsap IGKV4-1*01 F	91.92	Homsap IGKJ4*01 F	100.0	CQQYYSTPPTF
5 & 11	L101	IgL	Homsap IGLV1-47*01 F	94.29	Homsap IGLJ3*02 F	97.37	CAAWDDSMSDLNWVF
8 & 10 & 11	K60	IgK	Homsap IGKV4-1*01 F	91.25	Homsap IGKJ1*01 F	100.0	CQQYYGAPPWTF
8 & 11	K36	IgK	Homsap IGKV1-39*01 F	90.32	Homsap IGKJ5*01 F	94.74	CQQSYSNLAITF
8 & 11	K89	IgK	Homsap IGKV3-15*01 F	91.76	Homsap IGKJ2*01 F	92.11	CQHYYSRPPYNF
Single cell-sorted TT-LC cells							
14	45	IgK	Homsap IGKV1-12*01 F	94.27	Homsap IGKJ2*01 F	100.0	CQQTNSFPHTF
14	46	IgK	Homsap IGKV1-9*01 F	96.06	Homsap IGKJ4*01 F	94.44	CQQLHSYPRTF
14	51	IgK	Homsap IGKV2-28*01 F	96.6	Homsap IGKJ5*01 F	100.0	CMGGLQTPTF
14	5	IgL	Homsap IGLV1-40*01 F	100.0	Homsap IGLJ3*02 F	97.37	CQSYDSSLSGWVF
14	7	IgL	Homsap IGLV1-51*01 F	99.3	Homsap IGLJ3*02 F	100.0	CGTWDSSLRAWVF
14	6	IgL	Homsap IGLV1-51*01 F	96.84	Homsap IGLJ3*02 F	100.0	CGTWDNSLSPFWVF
14	3	IgL	Homsap IGLV2-23*02 F	92.71	Homsap IGLJ2*01 F	93.55	CSSYANASPSVVF
15	34	IgK	Homsap IGKV1-17*01 F	96.06	Homsap IGKJ2*01 F	100.0	CLQLNTYPYTF
15	41	IgK	Homsap IGKV1-5*03 F	94.27	Homsap IGKJ2*02 F	89.47	CQQYNSYPRTF
15	47	IgK	Homsap IGKV1-5*03 F	92.83	Homsap IGKJ4*01 F	94.74	CQQYNTYPLTF
15	39	IgK	Homsap IGKV1D-12*01 F	95.34	Homsap IGKJ2*01 F	94.74	CQQVDSFPHTF
15	20	IgK	Homsap IGKV3-20*01 F	91.84	Homsap IGKJ1*01 F	88.89	CQQFDITFWTF
15	1	IgL	Homsap IGLV2-8*01 F	95.49	Homsap IGLJ1*01 F	92.11	CSSYSGDNNYKYVF
15	56	IgL	Homsap IGLV3-19*01 F	93.19	Homsap IGLJ2*01 F	94.59	CNSRDNGGNHVIF
15	57	IgL	Homsap IGLV3-19*01 F	93.55	Homsap IGLJ2*01 F	94.59	CNSRDTTNNHVLF
17	32	IgK	Homsap IGKV1-17*01 F	94.98	Homsap IGKJ2*02 F	100.0	CLQHNSYPRTF
17	30	IgK	Homsap IGKV1-17*01 F	94.98	Homsap IGKJ2*02 F	100.0	CLQHNSYPRTF
17	31	IgK	Homsap IGKV1-17*01 F	94.98	Homsap IGKJ2*02 F	100.0	CLQHNSYPRTF
17	29	IgK	Homsap IGKV1-17*01 F	94.98	Homsap IGKJ2*02 F	100.0	CLQHNSYPRTF
17	49	IgK	Homsap IGKV1-39*01 F	89.96	Homsap IGKJ3*01 F	84.21	CQQSFTDSFDF
17	48	IgK	Homsap IGKV1-39*01 F	89.96	Homsap IGKJ2*03 F	92.31	CQQSYSTARFSF

Donor	Sequence	IgK/IgL	V-GENE and allele	V-REGION identity %	J-GENE and allele	J-REGION identity %	AA JUNCTION
Single cell-sorted TT-LC clones							
17	27	IgK	Homsap IGKV1-39*01 F	93.19	Homsap IGKJ3*01 F	92.11	CQQSDSAPFTF
17	28	IgK	Homsap IGKV1-39*01 F	97.13	Homsap IGKJ2*01 F	97.37	CQQSYSLPYTF
17	37	IgK	Homsap IGKV1-39*01 F	90.32	Homsap IGKJ2*01 F	94.74	CQQSHSTPYTF
17	52	IgK	Homsap IGKV1-6*01 F	94.62	Homsap IGKJ1*01 F	97.22	CLQHYNYPRTF
17	53	IgK	Homsap IGKV1-6*01 F	94.98	Homsap IGKJ1*01 F	100.0	CLQHYNYPRTF
17	24	IgK	Homsap IGKV3-20*01 F	92.55	Homsap IGKJ1*01 F	100.0	CRQYDAWPRTF
17	18	IgK	Homsap IGKV3-20*01 F	93.97	Homsap IGKJ5*01 F	92.11	CQQYGTSPRVPF
17	16	IgL	Homsap IGLV1-51*01 F	92.98	Homsap IGLJ2*01 F	86.21	CGTWDSSLSGVTF
17	15	IgL	Homsap IGLV1-51*01 F	92.98	Homsap IGLJ2*01 F	86.11	CGTWDSSLSGVTF
17	11	IgL	Homsap IGLV1-51*01 F	91.93	Homsap IGLJ2*01 F	83.33	CGTWDSSVNGVTF
17	12	IgL	Homsap IGLV1-51*01 F	92.98	Homsap IGLJ2*01 F	83.33	CGTWDSSLSGVTF
17	13	IgL	Homsap IGLV1-51*01 F	92.98	Homsap IGLJ2*01 F	83.33	CGTWDSSLSGVTF
17	14	IgL	Homsap IGLV1-51*01 F	92.98	Homsap IGLJ2*01 F	83.33	CGTWDSSLSGVTF
17	59	IgL	Homsap IGLV3-19*01 F	95.7	Homsap IGLJ2*01 F	88.89	CHSRDSSGNHRLF
23	33	IgK	Homsap IGKV1-33*01 F	99.64	Homsap IGKJ3*01 F	91.43	CQQYDNLPFF
23	36	IgK	Homsap IGKV1-33*01 F	94.62	Homsap IGKJ4*01 F	97.3	CQQYDHFITF
23	50	IgK	Homsap IGKV1-39*01 F	87.1	Homsap IGKJ2*01 F	97.37	CQQSYSSPYIF
23	38	IgK	Homsap IGKV1-39*01 F	94.98	Homsap IGKJ5*01 F	94.74	CQQSSSIPITF
23	35	IgK	Homsap IGKV1-39*01 F	87.1	Homsap IGKJ2*02 F	97.22	CQQNYDTPRTF
23	54	IgK	Homsap IGKV1-39*01 F	94.27	Homsap IGKJ1*01 F	97.22	CQQSYSTPRTF
23	44	IgK	Homsap IGKV1-5*03 F	95.7	Homsap IGKJ2*01 F	92.31	CQQYNSYPYTF
23	26	IgK	Homsap IGKV1-5*03 F	91.4	Homsap IGKJ1*01 F	86.84	CQHYDSPPWTF
23	43	IgK	Homsap IGKV1-5*03 F	91.76	Homsap IGKJ1*01 F	94.44	CQQYNSFSRTF
23	42	IgK	Homsap IGKV1-5*03 F	96.42	Homsap IGKJ1*01 F	97.14	CQQYNSYSTF
23	25	IgK	Homsap IGKV1-5*03 F	91.04	Homsap IGKJ1*01 F	94.59	CQHYDSPPWTF
23	40	IgK	Homsap IGKV1-5*03 F	94.27	Homsap IGKJ1*01 F	97.22	CQQYNSYSRTF
23	55	IgK	Homsap IGKV1-5*03 F	91.4	Homsap IGKJ1*01 F	86.84	CQQYDSPPWTF
23	17	IgK	Homsap IGKV3-15*01 F	99.28	Homsap IGKJ2*01 F	100.0	CQQYNNWPPYTF
23	23	IgK	Homsap IGKV3-20*01 F	94.62	Homsap IGKJ1*01 F	97.37	CQQYRSSPWTF
23	19	IgK	Homsap IGKV3-20*01 F	93.62	Homsap IGKJ3*01 F	97.37	CQQYTGSLFTF
23	22	IgK	Homsap IGKV3-20*01 F	96.81	Homsap IGKJ2*01 F	100.0	CQQSGGSPPYTF
23	21	IgK	Homsap IGKV3-20*01 F	93.97	Homsap IGKJ3*01 F	94.44	CQQYDSTALTF
23	8	IgL	Homsap IGLV1-51*02 F	92.63	Homsap IGLJ1*01 F	91.43	CGTWDSSLSVGVF
23	10	IgL	Homsap IGLV1-51*02 F	91.93	Homsap IGLJ1*01 F	91.43	CRTWDSSLSVGVF
23	9	IgL	Homsap IGLV1-51*02 F	92.63	Homsap IGLJ1*01 F	91.43	CGTWDSSLSVGVF
23	2	IgL	Homsap IGLV2-11*01 F	95.83	Homsap IGLJ2*01 F	85.71	CCSFAGGYTLLC
23	4	IgL	Homsap IGLV2-23*02 F	94.79	Homsap IGLJ2*01 F	94.74	CCSYAGNSNVVF
23	58	IgL	Homsap IGLV3-19*01 F	94.62	Homsap IGLJ2*01 F	91.43	CNSRDTGGDHRIF
